# A large-scale investigation of everyday moral dilemmas

**DOI:** 10.1093/pnasnexus/pgaf119

**Published:** 2025-05-13

**Authors:** Daniel Alexander Yudkin, Geoffrey Philip Goodwin, Andrew Reece, Kurt Gray, Sudeep Bhatia

**Affiliations:** More in Common, New York, NY 10003, USA; Wharton School, University of Pennsylvania, Philadelphia, PA 19104, USA; Department of Psychology, University of Pennsylvania, 3720 Walnut St, Philadelphia, PA 19104, USA; BetterUp Labs, Austin, TX 78702, USA; Department of Psychology and Neuroscience, University of North Carolina at Chapel Hill, Chapel Hill, NC 27599, USA; Department of Psychology, University of Pennsylvania, 3720 Walnut St, Philadelphia, PA 19104, USA

**Keywords:** morality, dilemmas, relationships, closeness, judgments, honesty, harm

## Abstract

Questions of right and wrong are central to daily life, yet scientific understanding of everyday moral dilemmas is limited. We conducted a data-driven analysis of these phenomena by combining state-of-the-art tools in machine learning with survey-based methods. We extracted and analyzed 369,161 descriptions (“posts”) and 11 M evaluations (“comments”) of dilemmas from the largest known online repository of everyday moral dilemmas: Reddit's “Am I the Asshole?” Users described a wide variety of everyday dilemmas on topics ranging from broken promises to privately held emotions. Dilemmas involving relational obligations were the most frequently reported, while those pertaining to honesty were the most frequently condemned. The types of dilemmas people experienced depended on the interpersonal closeness of the interactants, with some dilemmas (e.g. politeness) more prominent in distant–other interactions and others (e.g. relational transgressions) more prominent in close–other interactions. A preregistered follow-up investigation showed that similar dilemmas are reported in a census-stratified representative sample of the US population (*n* = 510). Overall, this paper highlights the diversity of moral dilemmas experienced in daily life and contributes to the development of a moral psychology grounded in the vagaries of everyday experience.

**Significance Statement**

People often wonder whether their previous actions were right or wrong. In this paper, we used a massive online repository of everyday moral situations, along with new methods in natural language processing, to investigate how people experience and evaluate moral dilemmas. Our results reveal 29 types of dilemmas, which vary in how frequently they are experienced and how negatively they are evaluated. While people most often experience dilemmas relating to relational obligations, they are most often condemned for acts of dishonesty. Furthermore, people experience different dilemmas depending on whether they are close or distant to whomever they are interacting with. Overall, these results underscore how relationships shape moral thinking and situate the study of moral psychology in everyday life.

## Introduction

In the course of daily life, people often face situations in which the right action is unclear. These moments represent everyday moral dilemmas: commonplace predicaments involving competing values and conflicting interpersonal obligations (1, 2). While science has discovered much about moral decision-making (3, 4), surprisingly little is known about everyday moral dilemmas. This is partly due to the methods employed in moral psychology, which typically ask participants to respond to hypothetical scenarios (5–7). By allowing researchers to systematically vary features of moral situations, these methods can shed light on how people process moral information (8). However, because they often focus on extreme or unusual cases (such as murder or incest (6, 9)), they risk neglecting the more mundane, but arguably more representative, moral questions that arise in daily life (10).

Moral dilemmas are defined in philosophical literature as situations in which an agent has “moral reasons to do each of two actions, but doing both actions is not possible” (11). Here, we adopt a broader definition drawn from the language of laypeople. We classify *everyday moral dilemmas* as “difficult situations” (12) arising in daily life in which the morally appropriate action is uncertain (2). Beyond uncertainty, these dilemmas share several other key characteristics. First, they tend to be *commonplace*, involving issues that people are likely to encounter in the course of normal life. Second, they are typically *mild*, involving events that are neither extreme nor even obviously immoral. Third, they often center on the *self.* People typically want to do the right thing (13, 14), and they typically ruminate on their own behavior when trying to adjudicate moral issues in daily life. Finally, because humans are social animals, everyday dilemmas tend to be *social*, concerning actions (and often conflicts) involving friends, family, and coworkers (15).

Past research has made some progress in understanding everyday moral dilemmas. For example, researchers have used ecological momentary assessment to explore how moral experiences affect happiness (16) and how the presence of others impacts the importance of moral values (17). Other work has explored the moral–psychological features of different corpuses of language, including from Twitter (18) and Facebook (19). Notably, however, all these studies relied on the moral foundations theory (MFT) taxonomy (20). This taxonomy, which identifies five (or, more recently six (21)) moral foundations, provides a theory-driven framework grounded in an evolutionary understanding of morality. This approach can help assess how morality varies across politics and culture; however, by focusing on a small number of moral concerns, it may overlook important distinctions in the nature of everyday moral experiences. In this paper, we sought to capture the complexity of morality in daily life by using a bottom-up (that is, data-driven) approach to map everyday moral dilemmas in the US population.

In study 1, we used natural language processing tools to extract and analyze a large-scale naturally occurring repository of everyday moral dilemmas: a forum on the online network Reddit called “Am I the Asshole?” (AITA; see method 1). Reddit is a decentralized online platform designed to facilitate user-driven discussions across a vast array of topics, organized into specialized communities (“subreddits”). We used qualitative and quantitative methods to create, test, and refine a list of the most commonly occurring dilemma types in a subset of these data (see Table 1) and then extrapolated this list to the full AITA dataset via supervised machine learning methods (methods 2–5). We used the resulting coded dataset to answer several questions about the nature of everyday English-language moral dilemmas (methods 6 and 7). In study 2, we sought to assess the generalizability of our findings with a preregistered study of a US census-stratified sample (method 8).

**Table 1.** List of dilemma types identified in the AITA dataset.

Moral theme	Dilemma type	Description
**Fairness and Proportionality**	*Procedural fairness*	Person A may not be adhering correctly to a principle or procedure
	*Distributive fairness*	Person A may not be making an appropriate allocation of resources, or be contributing their fair share
	*Reciprocal fairness*	Person A may be failing to sufficiently compensate person B for an initial act
	*Behavioral underreaction*	Person A may be underreacting in response to person B's behavior or predicament
	*Behavioral overreaction*	Person A may be overreacting in response to person B's behavior or predicament
**Feelings**	*Private emotion*	Person A may be experiencing an inappropriate or excessive emotion or desire that is unknown to others
	*Emotional underreaction*	Person A may be feeling or displaying too little of a desirable emotion
	*Emotional overreaction*	Person A may be displaying too much of an undesirable emotion
**Harm and Offense**	*Unintended harm*	Person A may be accidentally harming, offending, or otherwise negatively impacting person B, either through words or behavior
	*Intentional harm*	Person A may be purposely harming, offending, or otherwise negatively impacting person B, either through words or behavior
	*Allowed harm*	Person A may be failing to prevent harm, offense, or other negative consequences from befalling person B
	*Risked harm*	Person A may be risking causing harm, offense, or other negative consequences to person B
**Honesty**	*Concealment*	Person A may be concealing information
	*Revelation*	Person A may be causing harm, offense, or other negative emotions by telling the truth
	*Misrepresentation*	Person A may be being deliberately dishonest
	*Reporting to authority*	Person A may be reporting person B to authority
	*Not reporting to authority*	Person A may be declining to report person B to authority
	*Secret violation*	Person A may be divulging person B's secret
	*Cheating*	Person A may be cheating in a cooperative or trusting situation
**Relational Obligation**	*Relational omission*	Person A may not be performing a behavior that person B wants or expects them to do in the context of a relationship
	*Relational transgression*	Person A may be performing or seeking to perform a behavior that person B disapproves of in the context of a relationship
	*Relational demand*	Person A may be expecting person B to perform a given behavior, or feeling upset that behavior was not performed in the context of a relationship
	*Relational prohibition*	Person A may be attempting to prevent person B from performing a behavior in the context of a relationship
	*Broken promise*	Person A may be going back on a commitment
**Social Norms**	*Public transgression*	Person A may be publicly violating a social norm or convention
	*Stealing*	Person A may be taking something that does not belong to them
	*Privacy violation*	Person A may be violating person B's privacy
	*Impoliteness*	Person A may be being rude to person B
	*Judgmentalness*	Person A may be passing judgment on person B

On AITA, users post a paragraph describing a personal quandary they experienced, punctuated with the question “AITA?” (or occasionally “Would I be the asshole?”). Other users then comment on the original post with a moral judgment and an explanation of their reasoning. By convention, most commenters employ a common lexicon to indicate their opinions: “Not the asshole” (“NTA”) indicates exoneration; “You’re the asshole” (“YTA”) indicates condemnation. (“Everyone sucks here” [ESH] and “No assholes here” [NAH] are also possible, but less common.)

AITA is uniquely positioned to shed light on the nature of everyday moral dilemmas for several reasons. First, it is massive, containing hundreds of thousands of posts and millions of comments, with over 11 M users as of October 2023. While data size does not guarantee quality or representativeness, it does offer greater power to detect variation in even rarely occurring moral phenomena, which can be then be corroborated in a more representative sample. Second, it is theoretically meaningful, since the title question represents a colloquial way of interrogating whether or not one committed a moral infraction (see study 2 for confirmation of this assumption). Third, AITA's unique structure, in which moral evaluations for posts can readily be extracted from user comments, provides the opportunity to investigate not just what moral dilemmas people experience in daily life, but also how others evaluate these dilemmas.

Of course, exploring morality through AITA has some limitations. First, Reddit's user base skews younger (with 44% of the user base aged 18–29 years (22)), meaning that participant responses may not reflect those of humans in general, or even the whole US population. In an effort to address this issue, in study 2, we sought to corroborate our findings with a census-stratified representative sample of the US population, nevertheless, our results remain constrained to this group. A second concern is the nature of the dilemmas reflected in this forum. By framing moral questions around whether someone is “the asshole,” AITA posts may not encompass all moral concerns, such as those that are extreme or clearly wrong, and may bias users toward judgments of character rather than moral evaluations per se. However, its strength lies in accessibility—this informal, widely recognized phrasing encourages broad engagement with moral reflection in a way that more formal approaches may not. In study 2, we test and verify our assumption that AITA evaluations closely align with moral judgments.

Previous research has used topic modeling to explore language in the AITA repository (23). In that work, researchers identified five meta-categories in AITA posts—Identities, Aspects, Processes, Events, and Things—and found that particular topics (e.g. those concerning marriage) predict the presence of different moral foundations. The current paper extends that work in several ways. First, by testing the cooccurrence of general topics with preestablished moral topics, that previous study did not build a data-driven taxonomy of moral experiences (as we attempt to do here). Second, that study did not explore questions relating to how relationships and relational closeness may shape the experience of different moral dilemmas, nor how the prevalence of dilemmas changed over time. Thus, the current investigation differs from the previous one despite relying on a similar dataset.

Our analysis first sought to document the nature and prevalence of various everyday dilemmas. We thus tested the frequency with which the different dilemmas identified in our catalog appeared in the dataset and then tested which types of behaviors were most and least negatively evaluated. Next, we examined the context of everyday dilemmas by examining the relational contexts in which they tended to occur. To date, much research in moral psychology has focused on decisions involving “raceless, genderless strangers” (24). Yet a growing body of research highlights the important role that relationships play in shaping people's sense of moral obligation (15, 17, 25–27). Therefore, we sought to test how the relationships mentioned in posts moderated the frequency of different moral dilemmas. Of particular interest was the effect of *relational closeness*—that is, the interpersonal proximity of the interactants in the post. Research in fields including evolutionary biology (28), social psychology (29), and philosophy (30) suggests that relational closeness shapes moral thinking, with people showing more concern for close others. Our dataset provided us the opportunity to examine how everyday experiences differ according to whether one is interacting with close or distant others.

Altogether, this research aims to map the landscape of everyday moral dilemmas with new precision and to pave the way for additional conceptual, theoretical, and methodological advances in this area.

## Results

### Study 1

We used a combination of qualitative, quantitative, and machine learning methods to create, test, and refine a data-driven catalog of the most commonly occurring moral dilemmas in the AITA dataset (see methods 2 and 3). The human-coding phase of this process took over 1,300 participant-hours to complete. In order to render the catalog more comprehensible, we assigned the 29 resulting dilemma types to six distinct moral themes (see Table 1). These categories were intentionally created via thematic analysis rather than an analysis of underlying factor structure (see method 3). Factor analysis and other similar clustering methods are useful for clustering categories based on their cooccurrence, but our goal here was not to create superordinate categories of moral concern, as has been done in previous research (20, 21, 31). Instead, our aim was to group a large number of dilemma types into conceptually similar categories—a task that does not lend itself to correlational analysis. Consider *intentional harm* and *unintended harm*. We classify both as falling under the broader theme of Harm and Offense, despite the fact that moral scenarios involving intentional harm typically do not also involve unintentional harm. Because this categorization scheme was conceptual, rather than purely statistical, all primary analyses were conducted at the level of dilemma type (rather than moral theme), so they are not affected by this categorization scheme. Additional details regarding clustering-based approaches are given in SOM 3.1. Figure S8 displays a k-means cluster analysis of dilemma types. For clarity, we italicize the names of dilemma types (e.g. *secret violation*) and capitalize the names of moral themes (e.g. Honesty).

**Fig. 1.**
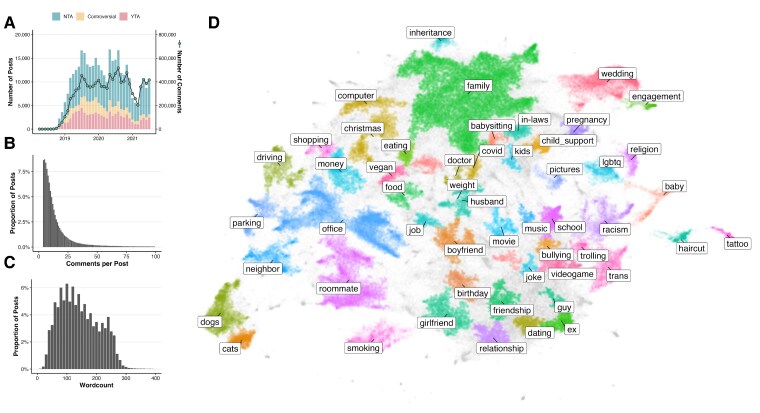
Overview of the AITA dataset (*n* = 369,161). A) Frequency of posts and comments across time. In this figure, posts whose negative evaluation score (weighted proportion of YTA + ESH comments) was less than one-third are labeled NTA; those whose negative evaluation score was greater than two-thirds are labeled as YTA; and those that did not reach either threshold are labeled as controversial. B) Frequency distribution of comments per post. C) Frequency distribution of word counts by post. D) A semantic map of the situations described across all posts. To create this visualization, we extracted an embedding (a 768-dimensional vector) of each post using the RoBERTa sentence encoder (32). We then reduced the embeddings to two dimensions and projected them onto a coordinate plane using UMAP (33). Then, we used the HDBSCAN (34) algorithm to identify clusters of posts and labeled each cluster according to its most prominent words (as reflected in an analysis of term frequency, inverse document frequency), “or TF-IDF.” The most frequently occurring situations pertained to family (21%), the office (8.4%), and weddings (4.2%) (35).

Pilot testing suggested that this catalog was comprehensive, accurate, and reliable (see Figs. S1–S3). We then used supervised machine learning to extrapolate from a subset of human-labeled AITA posts (*n* = 5,090) and assign dilemma types to each post in the full dataset (*n* = 369,161; see methods 4 and 5). Descriptive characteristics of this dataset are displayed in Fig. 1. Robustness tests suggested that our algorithm achieved a level of accuracy rivaling that of a human benchmark (see method 5 and Fig. S5). The result was a large dataset consisting of algorithmically generated predictions of the extent to which each dilemma type was present in each post (a measure we refer to as “prevalence”). We also translated prevalence values into binary values reflecting the model's best guess as to whether or not each post contained each dilemma type (see method 5). Note that each post could reflect multiple dilemma types. We base all primary study 1 analyses on these datasets.

#### Content analysis

**Fig. 2.**
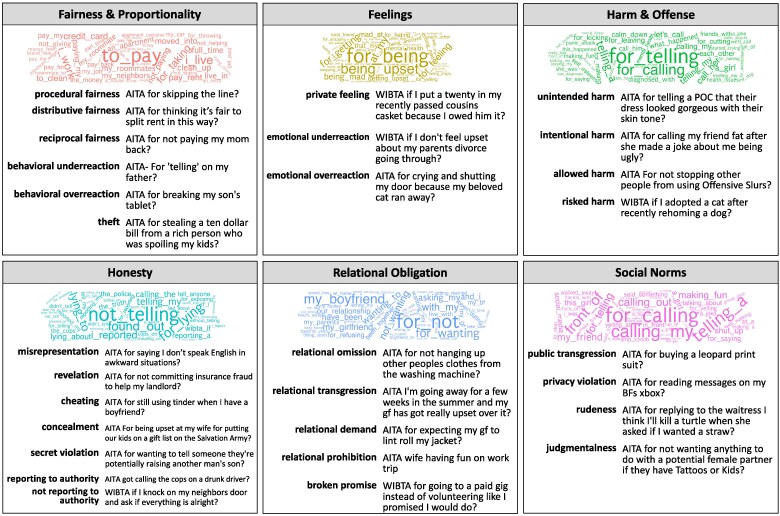
Overview of the 29 most common dilemma types present in the AITA dataset, grouped according to moral theme (*n* = 369,161 posts). Word clouds reflect the bigrams with the highest relative frequency for each moral theme. The name of each dilemma type is displayed along with the title of that dilemma type's most “exemplary” post (i.e. the post for which that dilemma type was most prevalent). The fact that the post titles aptly characterize their corresponding dilemma type supports the claim that our algorithmic approach was successful in detecting subtle differences in moral language.

Before analyzing the newly created dataset, we conducted an exploratory text analysis of the language in each moral theme. We first examined the most commonly occurring bigrams (i.e. two-word phrases) within each moral theme (see Fig. 2). The results highlight the everyday concepts reflecting each theme. For example, the most common bigrams in posts pertaining to Fairness and Proportionality included “to pay” and “for taking”; to Honesty, “not telling” and “for lying”; and to Feelings, “for being” and “being upset.” These findings suggest that the model is identifying expected moral content in the dataset and highlight the topics reflected in each theme.

To better understand the everyday situations reflecting each dilemma type, we next examined the title of the best-fitting post for each dilemma type (as reflected in the prevalence scores; see Fig. 2). The results provide a detailed picture of the moral dilemmas people experience in daily life. Consider the three types of fairness identified in the dilemma catalog: *procedural, distributive,* and *reciprocal*. The catalog defines these dilemma types as someone “not adhering correctly to a principle or procedure,” “not making an appropriate allocation of resources,” and “not sufficiently compensating[someone] for an initial act,” respectively. If our algorithm is detecting legitimate differences between these dilemma types, then the exemplars of each category should reflect these definitions. Indeed, we find that the best-fitting post for *procedural fairness* concerns questions about skipping the line, for *distributive fairness*, questions about splitting the rent, and for *reciprocal fairness*, questions about reimbursing one's mother. Similar distinctions are observed in the Harm and Offense theme, which includes four types: *unintended*, *intentional*, *allowed*, and *risked*. Accordingly, we observe in the strongest exemplar of the first category a reference to an inadvertently insensitive remark; in the second, a deliberate insult; in the third, the failure to prevent others’ “offensive slurs”; and in the fourth, a risk of harm to a pet. The results of this analysis suggest that the catalog is making meaningful and intuitive distinctions between different dilemma types and that the algorithmic process that we used to identify different dilemma types is capturing these distinctions.

#### Frequency analysis

**Fig. 3.**
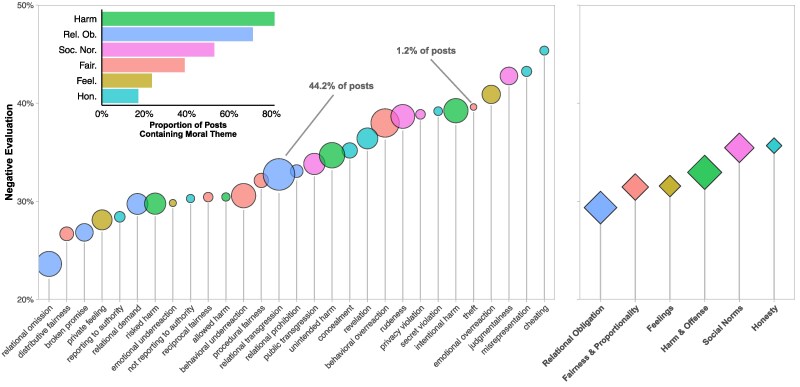
Average negative evaluation of each of the 29 dilemma types and six moral themes identified in the AITA catalog of everyday dilemmas (*n* = 369,161). In the main panel, dilemma types are ordered according to evaluation; dot size corresponds to relative frequency of occurrence of each dilemma type across all posts with range (1.2%, 44.2%). As shown in the figure, several forms of dishonesty, including cheating, misrepresentation, and secret violation, are among the most negatively evaluated dilemma types; the most common dilemma types are various forms of relational obligation as well as behavioral overreaction. In the right panel, moral themes are similarly plotted according to frequency and evaluation. The inlaid panel on the top left indicates the frequency of occurrence of the different moral themes.

Having validated our methods, we next investigated which moral dilemmas users reported most frequently. Our analysis suggests that the landscape of everyday moral dilemmas is broad and multifaceted: of the 29 dilemma types identified in the list of everyday dilemmas, almost half (13) appeared in more than 10% of posts. Two of the most frequently reported dilemma types were *relational transgression* (44.2% of posts) and *relational omission* (24.0% of posts), highlighting the potential importance of relational obligations in everyday moral experience. *Behavioral overreaction* and *unintended harm* were also common (35.7 and 22.9%, respectively; see Fig. 3). In addition, several dilemma types that are underrepresented in moral psychology occurred with notable frequency. These include *revelation* (i.e. telling a potentially hurtful truth, 14.3% of posts), experiencing a (potentially objectionable) *private feeling* (12.7% of posts), *rudeness* (21.2% of posts), *broken promise* (9.0% of posts), *judgmentalness* (8.7% of posts), and *privacy violation* (2.3% of posts).

These findings have several implications. First, the prevalence of dilemmas concerning relational obligations suggests that questions pertaining to one's responsibilities in different relationships feature prominently in daily life. While these types of dilemmas are conceptually related to the moral foundation of “loyalty” (20), they also differ from loyalty in important ways (see method 3). Second, these results highlight the diversity of everyday moral dilemmas, many of which remain poorly understood. For example, while past research has explored the *private feelings* that arise in *response* to various moral transgressions (36), it has not yet extensively explored emotions as *objects* of moral evaluation unto themselves (i.e. “Is it wrong to feel this way?”) (37, 38). Similarly, while recent research has explored how people perceive communicators who consider the social consequences of being honest (39) (see also refs. (40, 41)), important questions remain about how they evaluate potentially hurtful instances of truth-telling (*revelation*). Other underresearched topics include how people calibrate their reaction to others’ behavior, how they evaluate broken promises, how they consider violations of privacy, how they evaluate instances of risked versus actual harm, how they think about instances of judgmentalness, and how they experience, identify, and evaluate instances of impolite behavior (though see refs. (41–44) for initial research in these areas). In sum, our results show that users are commonly reporting moral experiences about which little is known in moral psychology.

#### Relationship to MFT

We next tested whether the dilemma types we identified could be captured by the moral concerns identified in MFT (20, 21). To do this, we first extracted the relevant moral dimensions from each post in the principal dataset using the extended Moral Foundations Dictionary (45). We then tested for correlations between the prevalence in each post of each dilemma type in our catalog and the prevalence of each moral foundation. The full results are presented in SOM 3.2 and Fig. S9. In brief, we observe many expected correlations. For example, *distributive*, *reciprocal*, and *procedural* fairness are all most strongly correlated with the Fairness foundation (*r* = 0.29, *r* = 0.26, and *r* = 0.20, respectively), while *allowed harm* and *risked harm* are both most strongly correlated with the Harm foundation (*r* = 0.17 and *r* = 0.14, respectively). This suggests that in certain key respects, both taxonomies are detecting convergent moral information. However, other everyday dilemmas have no notable correlations with moral foundations. This includes behaviors such as *misrepresentation*, *private feeling*, *privacy violation*, *relational omission*, *relational demand*, and *secret violation* (all *r*s < 0.1). This suggests that variation in everyday moral experiences is not reducible to the values reflected in MFT.

#### Moral evaluation

We next tested how users evaluate everyday moral behaviors. For this analysis, we included only those posts in which the post author was identified as the person who committed the act in question (89.0% of the sample), thereby ensuring that respondents’ evaluations were directed toward the perpetrator of the act rather than the recipient. We compared average rates of negative evaluation (that is, the weighted proportion of YTA and ESH votes relative to all verdicts) across dilemma types (see Fig. 3). The average rate of negative evaluation across posts was 31%. In the following analyses, all differences are significant at *P* < 0.001, Bonferroni-corrected; we report means and 95% CIs for each result.

Through this method we were first able to recover several well-established findings in psychological science. For example, replicating past research (46), *unintended harms* were evaluated less negatively than *intentional harms* (*M* = 34.7 ± 0.26 versus *M* = 39.2 ± 0.29%, *P* < 0.001, **d** = 0.12). Also consistent with past work (47), acts of *concealment* were evaluated less negatively than *misrepresentation* (being deliberately dishonest; *M* = 35.1 ± 0.51 versus *M* = 43.2 ± 0.92%, *P* < 0.001, **d** = −0.21). In addition, consistent with research showing that acts of omission are judged less harshly than acts of commission (48, 49), *relational transgressions* (performing unwanted behavior) were evaluated more negatively (*M* = 32.7 ± 0.19%) than *relational omissions* (failing to perform desired behavior) (*M* = 23.6 ± 0.23%, *P* < 0.001, **d** = −0.25). Similarly, cases of *underreaction* (displaying too little of a desirable behavior) were evaluated less negatively (*M* = 30.6 ± 0.27%) than cases of *overreaction* (displaying too much of an undesirable one; *M* = 38.0 ± 0.23%, *P* < 0.001, **d** = 0.19). These findings suggest that patterns of evaluation in the dataset reflect established differences in moral assessments.

Next, we examined which dilemma types were evaluated most negatively. The results showed that the two most negatively evaluated dilemmas pertained to acts of dishonesty, namely *cheating* (cheating in a cooperative or trusting situation; *M* = 45.3 ± 1.0%) and *misrepresentation* (*M* = 43.2 ± 0.92%). These were evaluated significantly more negatively than dilemmas involving *intentional harm* (*M* = 39.2 ± 0.28%): *P* < 0.001, **d** = 0.15 and *P* < 0.001, **d** = 0.10, respectively. This suggests that violations of trust were, on average, evaluated more negatively than acts of harm. Corroborating this notion, the moral theme of Honesty was evaluated more negatively than that of Harm and Offense (*M* = 35.7 ± 0.001 versus *M* = 33.0 ± 0.001%, *P* < 0.001, **d** = 0.07).

Unexpectedly, the third most negatively evaluated dilemma type pertained neither to harm nor honesty, but rather to *judgmentalness* (defined as passing judgment on someone, *M* = 42.7 ± 0.44%). This finding, which is consistent with recent work (50), suggests that the act of imposing a moral evaluation on others can itself be considered morally wrong, perhaps by eliciting perceptions of self-righteousness or hypocrisy (51).

We next examined those dilemma types eliciting the *least* negative evaluation. We anticipated that dilemmas concerning *private feelings* would be evaluated the least negatively, since they would not be perceived as causing negative outcomes for others. Yet, while these were among the least negatively evaluated (*M* = 28.1 ± 0.32%), the least negatively evaluated dilemma type (by a wide margin) was *relational omissions* (*M* = 23.6 ± 0.23%). The idea that relational obligations are less likely than other dilemma types to elicit negative evaluations is corroborated by the fact that Relational Obligations were the least negatively evaluated moral theme (*M* = 29.4 ± 0.001%); the second-least negatively evaluated moral theme was Feelings (*M* = 31.6 ± 0.001%, *P* < 0.001, **d** = 0.06).

Several conclusions emerge from these observations. First, the fact that many honesty violations were evaluated so negatively—even more than intentional harm violations—is consistent with theorizing suggesting that people are highly sensitive to violations of trust, possibly because such defections can undermine long-term cooperation (52). Of course, because everyday moral dilemmas are uncertain, it is likely that egregious instances of harm are excluded from this dataset, and we cannot say that honesty dilemmas would be judged more negatively once controlling for certainty or severity. Nevertheless, these observations provide a notable, albeit qualified, piece of evidence underscoring the importance of honesty in moral life. Second, the findings pertaining to *relational omission* suggest that, while not complying with others’ wishes (a behavior known in clinical settings as “setting boundaries,” (53)) represents a frequent source of moral doubt, it is not typically penalized by outside observers. In other words, people may be more concerned about the negative ramifications of setting boundaries than they need to be.

#### Relational context

**Fig. 4.**
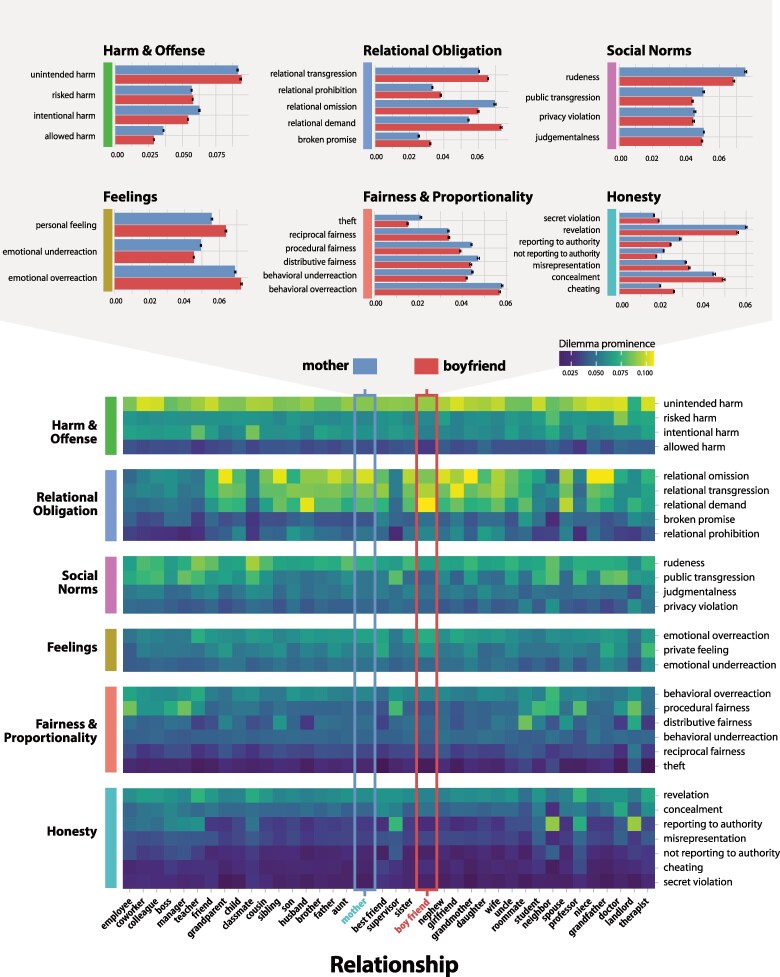
Prevalence of each dilemma type in each relational context. For this analysis, we used only the posts (*n* = 298,632, or 80.1% of data) that contained a common and identifiable relationship (see method 6 for additional details). Prevalence values reflect the mean predicted likelihood of each dilemma type appearing in posts containing each relational context (theoretical range: [0, 1], *M* = 0.051, SD = 0.020). Bar graphs provide example comparisons of prevalence values between the *mother* and *boyfriend* relational context; error bars = 95% CI.

We next examined which dilemma types appeared in different relational contexts. We used automated text analysis to detect which relationship categories (if any) were referenced in each post and then computed the average prevalence of each dilemma type in each relational context (see Fig. S6 for relationship frequencies and method 6 for additional details). The results form a 41 × 29 matrix showing the likelihood of each moral dilemma occurring in each relationship (Fig. 4).

An inspection of this matrix illustrates the complex ways that relationships modulate everyday moral experiences. To illustrate, consider two of the most frequently occurring relational contexts: *boyfriend* (*n* = 28,381) and *mother* (*n* = 28,533). Dilemmas involving *cheating* were more likely to occur with boyfriends than with mothers, *t*(56,912) = 48.14, *P* < 0.001, **d** = 0.40, while those involving *reporting to authority* were more likely to occur with mothers than with boyfriends, *t*(56,912) = −22.28, *P* < 0.001, **d** = −0.19. At the same time, while dilemmas involving *relational omission* (failing to do what is expected) were more prevalent with mothers, *relational demand* (expecting someone else to perform a behavior) were more likely with boyfriends, *t*(56,912) = 57.54, *P* < 0.001, **d** = 0.40—thereby illustrating a widely recognized trope that people tend to disappoint their mothers but be disappointed by their boyfriends. Starker distinctions in the prevalence of different dilemma types emerge when more dissimilar relational contexts are compared (e.g. *sister* and *manager*; see Fig. 4).

While these differences may match commonsense expectations, other arguably more revealing findings also emerge. For example, cases of *judgmentalness* are more common when one is interacting with one's *wife* than one's *husband*, *t*(26,396) = −10.95, *P* < 0.001, **d** = −0.14; cases of *unintended harm* are more common when one is interacting with one's *daughter* than one's *son*, *t*(11,497) = 8.3, *P* < 0.001, **d** = 0.15; and cases of *risked harm* are more common when interacting with one's *doctor* than one's *therapist*, *t*(706) = 6.36, *P* < 0.001, **d** = 0.47. Overall, these data offer a generative template for investigating how different relational contexts give rise to different moral dilemmas. A user-friendly interface to explore the data is available at https://danielyudkin.shinyapps.io/everyday-moral-dilemmas. In SOM 3.3, we examine associations between dilemma types and another framework that seeks to explain how relationships modulate morality, relational models theory (15). Overall, these findings offer a precise picture of how relationships impact moral experience.

#### Relational closeness

Having explored how different moral dilemmas are reported in different relational contexts, we next sought to test the extent to which relational closeness could explain these differences. We focused on this dimension given its central role human relationships (54) and theoretical relevance in fields such as theoretical biology (e.g. via kin selection (28)), social psychology (e.g. via group affiliation (29)), and philosophy (e.g. via the moral circle (30)). To assess relational closeness in each post, we extracted all common relationship words across all posts and asked a separate sample of online workers to indicate how close that relationship was, on average (see method 7). Based on previous research suggesting that values such as loyalty are activated in the presence of close others (17), we anticipated that different forms of relational obligation (e.g. *prohibition*, *demand*, *transgression*, and *omission*) would be more prevalent in close relationships. By contrast, *public transgressions* should (by definition) occur less frequently in interactions with close others. Moreover, in light of findings suggesting that politeness serves to “reflect and regulate social distance,” (55, 56) we expected dilemmas involving *rudeness* also to be more prevalent among distant others.

**Fig. 5.**
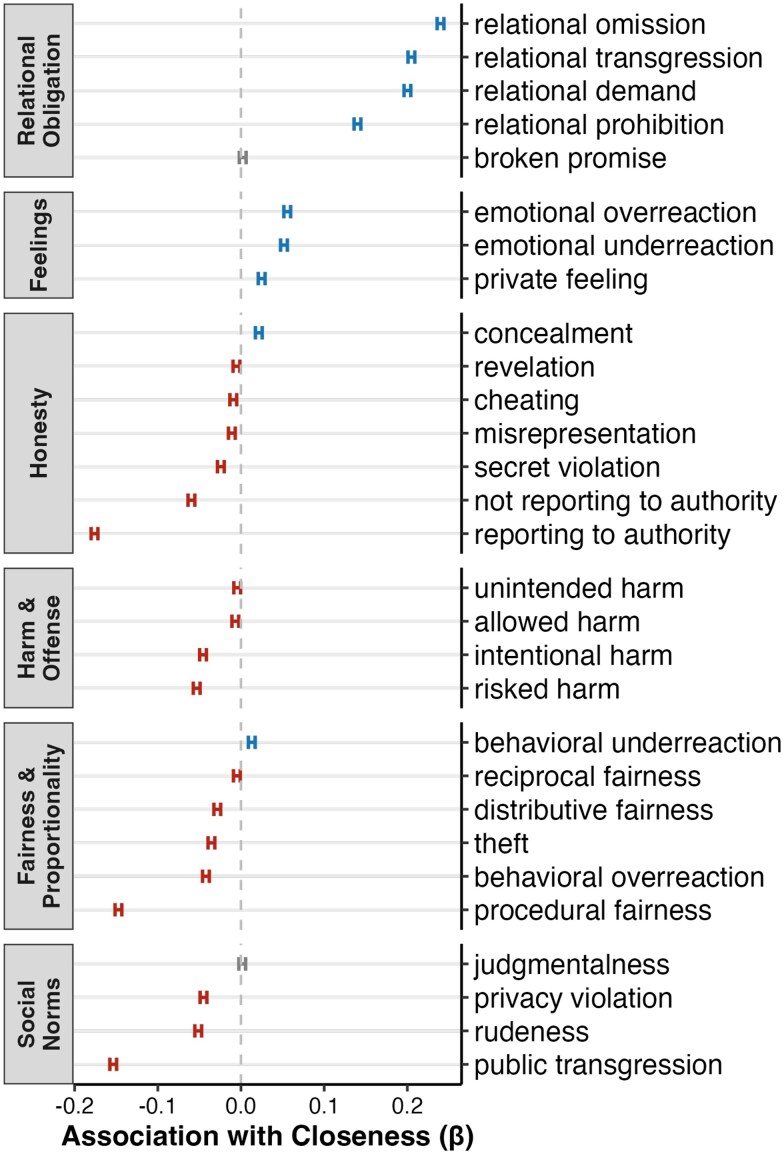
Associations between the prevalence of each dilemma type and the estimated interpersonal closeness of the interactants. Intervals = 95% CIs around standardized beta coefficients. Estimates colored red are significantly below zero, blue significantly above zero, and gray not significantly different from zero.

As predicted, the likelihood of experiencing a dilemma related to *relational omission* (*β* = 0.23)*, relational transgression* (*β* = 0.21), *relational demand* (*β* = 0.20), and *relational prohibition* (*β* = 0.13) was, in each instance, positively associated with relational closeness (Fig. 5). (In these results, 95% CIs containing all reported standardized betas were <0.005.) Conversely, strong negative associations emerged between closeness and *public transgression* (*β* = −0.15) and *rudeness* (*β* = −0.05). Two other dilemma types showed robust negative associations with closeness. The first was between closeness and *reporting to authority* (*β* = −0.18)—an observation that is consistent with past findings showing that people enforce social norms (i.e. by appealing to authority) more frequently when the putative transgressor is an outgroup versus an ingroup member (57). The second was between closeness and *procedural fairness* (*β* = −0.15), a finding that accords with the idea that following correct procedures can help regulate interactions between distant others.

These findings are notable in light of past research, which suggests that people show greater moral concern for close others (30). Here, by contrast, we find that, while certain moral dilemmas are more frequently experienced among close others, other dilemmas are more frequently experienced among distant others. This suggests that the manner in which closeness modulates everyday moral experiences is not unidirectional. Instead, our data are consistent with an account in which moral concerns are strategically deployed to regulate behavior vis-à-vis close or distant others depending on the demands of the situation.

### Study 2

Our analysis of AITA suggests the existence of many commonly experienced but still unexplored everyday moral dilemmas. However, Reddit users may not be representative of the population. Accordingly, to explore the generalizability of our findings, we conducted a preregistered follow-up study using a census-stratified representative sample of the US population collected from an online worker platform. Participants (*n* = 510) described a “personal experience you've had that caused you to worry about whether you were in the wrong” (see SOM 2.2 for full instructions). These dilemmas were then evaluated by a separate sample of participants and coded according to the same dilemma catalog developed in study 1.

The study was designed to address several unanswered questions in study 1. First, specific features of the AITA context, including idiosyncrasies of the user base and norms on the forum, might select for certain kinds of moral dilemmas over others. By asking our panel to describe a time in which they worried whether they were “in the wrong,” we ensured that the participants’ descriptions would reflect the broad scope of everyday moral dilemmas. Second, while we have suggested that the title question of AITA is a colloquial way of asking whether someone is in the wrong, we have not yet provided empirical support for this claim. For example, the term “asshole” indicates a character judgment, while wrongness pertains to an act itself. Thus, it remains unclear the extent to which descriptions conveyed in the AITA dataset reflect moral dilemmas more generally. For this reason, we also asked evaluators of the moral dilemmas to indicate the extent to which they believed the writer of the story was “in the wrong.” This allowed us to ensure that evaluations of these dilemmas reflect moral judgments per se.

Our preregistered analyses sought, first, to verify that our established catalog of moral dilemma types was adequately capturing the dilemmas described in this representative sample. Next, we tested the correlation between the frequency of dilemma types obtained in the AITA sample versus the representative sample. This allowed us to determine the extent to which the dilemma types in AITA were reflected in the broader population. In exploratory analyses, we then examined the correlation between the evaluations of the dilemmas in both datasets. We focus here on the correlation analyses; additional analyses (all of which confirm our preregistered predictions) are found in method 8.

**Fig. 6.**
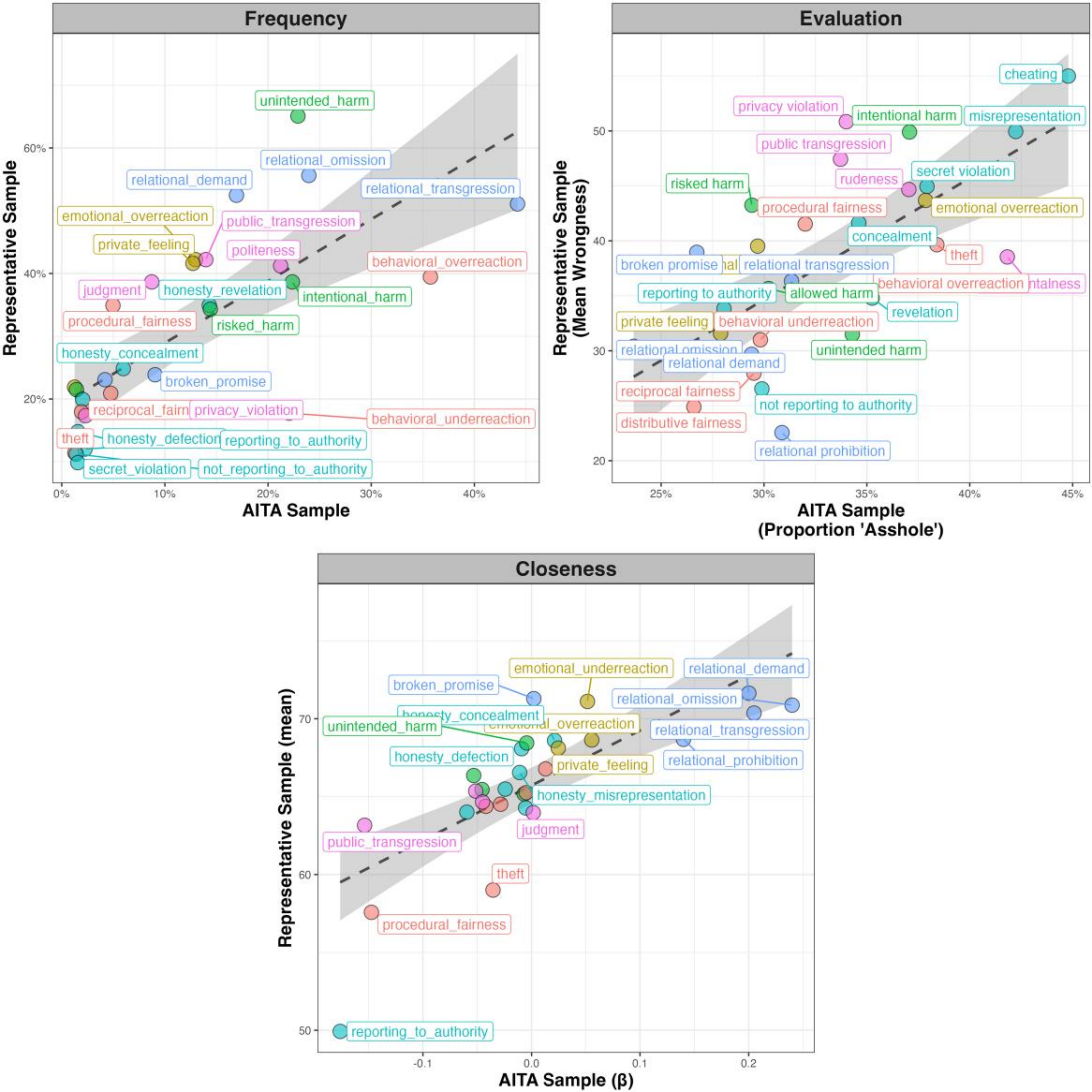
Correlations between features of dilemma types extracted from the AITA database and those observed in the representative sample (study 2). The top left panel shows the relationship between the frequency of the various dilemma types in the AITA sample (*n* = 369,161) and that in the representative sample (*n* = 510), *r*(27) = 0.73. The top right panel shows the relationship between the average negative evaluation (that is, proportion rated “YTA”) of dilemmas in the AITA sample and the average wrongness judgments of dilemma in the representative sample, *r*(27) = 0.68. The bottom panel shows the correlation between the beta weight reflecting the association between dilemma prevalence and relational closeness in the AITA sample, and the average closeness of the relational contexts in which the various dilemma types occurred in the representative sample, *r*(27) = 0.75.

Our analyses revealed robust correlations (Fig. 6). Most critically, there was a strong correlation between the frequency of dilemma types in the AITA dataset as compared to the representative sample, *r*(27) = 0.73, *P* < 0.001. This shows that the specific dilemma types that occur commonly in AITA also appear to occur in the daily lives of our representative sample. There are a few additional similarities and differences between the datasets worth noting. One difference is that in the AITA sample, the most commonly occurring dilemma was *relational transgression*, while in the representative sample, it was *unintended harm*. A similarity is that *relational demand*, *relational omission*, and *relational transgression* were three of the top four most commonly appearing dilemma types in both datasets. Furthermore, the same unexplored dilemmas that we observed in study 1—namely, *privacy violation, rudeness*, *judgmentalness*, *behavioral overreaction*, *private feeling*, and *broken promise*—all occurred in the representative sample at frequencies >10%, confirming that these are widely encountered experiences. More generally, these analyses suggest that the dilemmas contained in the AITA dataset reflect those encountered in real life.

Next, we evaluated the association between negative evaluations of dilemma types obtained from the AITA sample and those obtained from the representative sample. Again, analysis revealed a strong correlation, *r*(27) = 0.68, *P* < 0.001. This suggests that negative ratings in the AITA data reflect real-life wrongness judgments. Finally, we sought to corroborate the findings demonstrating divergent associations between relational closeness and the prevalence of different everyday dilemmas. To do this, we tested the correlation between the beta weights reflecting the association between dilemma prevalence and closeness obtained from study 1 with the average relational closeness associated each dilemma in the study 2 dataset. The results revealed a robust correlation, *r*(27) = 0.75. This corroborates the findings in study 1 showing how relational closeness moderates the occurrence of different dilemma types in daily life.

## Discussion

Everyday moral dilemmas have occupied a central place in human discourse across culture and time. In Plato's *Republic*, for example, Socrates contemplates the appropriateness of returning a borrowed weapon to a friend who is not in his right mind (an example of *relational omission*?). Our findings advance understanding of these dilemmas in a number of ways. First, they highlight the sheer diversity of everyday moral experiences. Participants in our dataset grappled with a wide variety of moral questions, ranging from the appropriateness of one's feelings about a divorce to the fairest way to split the rent. Many efforts have been made to reduce the moral landscape to a handful of categories—efforts that, as with the creation of our “moral themes,” can help make this landscape more navigable. Yet, our research suggests that there may be times in which a more granular approach is preferable for capturing the complexity of moral life.

These findings also highlight the degree to which social relationships impact everyday moral experiences. First, we find that the most frequently occurring type of moral dilemma concerns relational obligations. Not only that, we find that the kind of dilemmas people experience vary widely depending on whom they are with. Thus far, researchers in moral psychology have tended to shy away from studying the effect of particular relationships on moral judgments, opting instead to study depersonalized contexts that leave the precise relationship between actors unspecified. The rationale for this approach is that omitting the “noise” of relational contexts allows for a more precise understanding of moral cognition. Yet, our data suggest that this approach may be throwing the baby out with the bathwater: of the thousands of dilemmas contained in our dataset, over 80% involved an identifiable relationship between actors. This suggests that experimental strategies in moral psychology that omit relational information may be missing out on a constitutive feature of moral cognition. In sum, our research adds to a growing body of work suggesting that morality may be thought of less as a set of abstract principles and more as a “relational toolkit,” guiding and constraining behavior according to the demands of the social situation.

Our results also underscore the importance of honesty in everyday morality. Evolutionary psychologists have long argued that humans consider acts of dishonesty among the most egregious moral violations, since such acts threaten group cooperation. For example, some researchers have posited that humans possess a “cheater detection mechanism” that is particularly attuned to perceived betrayals (52). Evidence for this has come from a variety of sources, but little from everyday moral experiences. In our data, not only were honesty-related dilemmas fairly common, several forms of dishonesty (i.e. cheating, lying, and secret violation) were among the most negatively evaluated behaviors—more so even than acts of intentional harm. This is thus consistent with the perspective that people may be particularly vigilant to violations of honesty, possibly because such violations undermine cooperation.

Our paper adds to a growing field using advanced digital methods to explore psychological phenomena. AI models have been used to code and interpret a number complex psychological variables, including personality, implicit biases, decision-making, health, risk perception, consumer behavior, and moral behavior (58–61). Here, we leveraged methods in AI to create a rich dataset containing descriptions and evaluations of everyday dilemmas, along with indications of the dilemma types present in each description. Our dataset could be used to answer any number of additional questions that were not addressed in this paper, ranging from the effect of gender to the prevalence of different emotions (e.g. jealousy, anger, frustration, etc.) to the impact of the use of certain words (e.g. “obligation”) on users’ evaluations. This dataset, which contains unique post identifiers that can be matched to current posts on Reddit, may facilitate additional investigations into the nature of everyday moral experiences.

Despite its strengths, our approach has important limitations. First, because it focuses on the complex, mundane, and often uncertain subject matter of everyday life, it does not offer a comprehensive model of how people deal with severe moral violations, or bizarre or unusual cases that test the boundaries of our moral intuitions. Second, as noted in our introduction, while our dataset does provide evidence for the types of dilemmas experienced in the US context, there is evidence that moral experiences are very different outside the White, Educated, Industrialized, Rich, and Democratic context (21, 62). Collectivist societies, which emphasize group harmony, social obligation, and relational interdependence (63), may approach moral dilemmas differently. For instance, while Western moral judgments often prioritize individual rights and fairness, collectivist cultures may place greater emphasis on relational harmony and filial piety. If anything, this would imply that everyday dilemmas in those contexts would emphasize relational obligations even *more* than this sample does, although future research would be needed to test this assumption. In sum, while the everyday moral dilemmas identified in our analysis do not represent an exhaustive list of the kinds of dilemmas experienced by all humans around the world, they provide a broader and clearer picture of the landscape of English-language everyday moral experiences than has thus been attained in psychological science.

Disagreements over right and wrong form the basis of conflicts ranging from marital spats to civil war. While descriptions of large-scale conflicts tend to occupy our newspapers and airwaves, our findings suggest that many of the moral questions people face concern the messy, quirky web of responsibilities they have to the people in their lives. Developing a more nuanced understanding that reflects these responsibilities may not only advance theories in moral psychology and philosophy, but could also help mitigate interpersonal disagreement by identifying and diffusing sources of moral conflict (64). In this way, it could contribute to efforts aimed at improving everyday moral life.

## Materials and methods

### Method 1: Data extraction

Our first step was to extract the AITA data, sort it, clean it, and render it usable for analysis. We used Pushshift, an application programming interface (API) widely used in data science for accessing the Reddit database (65) to extract the data. Pushshift conforms with Reddit's terms of service, and our study was approved by the Institutional Review Board (IRB) at the University of Pennsylvania. We extracted a total of 509,230 posts and 17,822,228 comments associated with the AITA subreddit. Of these, in order to ensure post quality and engagement (since posts with very low engagement are typically of low quality), and in line with previous research (23), we selected for inclusion only those posts that had received at least five comments and only those comments that contained an explicit judgment (NTA, YTA, NAH, and ESH).

After extraction, filtering, and cleaning, our principal dataset comprised 369,161 posts and 11,280,903 comments ranging in time from 2018 April 4 to 2021 July 25. We next created an average negative evaluation for each post. In line with previous research (23), we calculated this rating by weighting each comment by its number of upvotes and computing the proportion of all weighted comments containing a negative evaluation (i.e. “YTA” or “ESH”). Upvotes provide users the chance to endorse comments and posts on Reddit by clicking a “thumbs up” icon adjacent to the text. The result was an average negative evaluation for each post ranging from 0 to 100%. We used this “weighted” method for calculating negative evaluation because the majority of users indicate their verdict by upvoting comments rather than leaving a comment themselves; thus, this approach arguably captures more information regarding users’ attitudes toward posts. However, we note that negative evaluations computed via the “unweighted” approach (in which comments are tallied without considering their upvote count) are correlated with the weighted approach at *r* = 0.96, and employing this unweighted approach produces no substantive difference to our results.

### Method 2: Construction and validation of the AITA dilemma catalog (“pilot study”)

In order to identify the dilemmas present in the AITA dataset, we relied on a three-step process consisting of qualitative methods, then quantitative methods, and then finally machine learning. First, it was necessary to identify the moral topics reflected in the data. While several taxonomies already exist that can identify moral themes in language (45, 66), the purpose of our study was to generate a catalog of moral dilemmas in a “bottom-up” (that is, data-driven) manner. We thus relied on qualitative methods to identify the initial list. The qualitative stage followed principles of Descriptive Coding, a method that allows ethnographers to identify recurring themes in written or spoken text (67).We employed a three-step process using qualitative methods to identify and categorize moral dilemmas systematically.

1. **Domain identification**: The first, second, and last authors began by analyzing a randomly selected subset of ∼400 posts. Through iterative coding, we identified key categories of moral dilemmas. We then generated *category* labels, *definitions*, and *examples* of each dilemma category.
2. **Refinement and expansion**: Next, we independently coded an additional sample of 100 posts to test and expand the taxonomy. Coding focused on identifying features that distinguished each moral domain. Disagreements were resolved through dialogue, and novel terms were integrated into the evolving taxonomy.
3. **Catalog production**: We iteratively refined the list to accommodate novel posts and to generate final names and descriptions of the dilemma types comprehensible to human coders. The results produced a preliminary catalogue of 29 dilemma types that could be subjected to pilot testing.

We then conducted a pilot test to determine whether this initial list was achieving adequate descriptive validity of AITA posts—that is, whether the dilemma categories consistently tracked ordinary people's understanding of what the posted dilemmas were about. We recruited 79 undergraduates (28 men, 49 women, and 2 other, *M* age = 19.9, SD = 1.02) from a large university in the northeastern United States to participate in a 30-min. study in exchange for course credit. The study was determined to meet eligibility criteria for IRB review exemption by the IRB at the University of Pennsylvania, protocol #823184. After providing informed consent, reading full instructions introducing the dilemma types, and successfully completing a series of comprehension checks, participants were presented with 10 posts chosen from a randomly selected subset of 300 posts. For each post, participants were asked to provide several responses. First, they were asked which of the 29 dilemma types described what was occurring in the post. They were offered the opportunity to select as many applied, as well as the chance to indicate “other” (and provide a free response) if they believed none of the experimenter-supplied dilemmas described the post. Next, for each of the dilemma types they had selected, they indicated how well that dilemma type “fit” (that is, described) the post (1—“Not at all well” and 5—“Extremely well”). This was termed “fit score.” Next, they indicated whether the writer of the post was the perpetrator or the recipient of the act in question. Finally, they provided their own evaluation of the post in question (e.g. NTA/YTA).

#### Pilot 1 results

Participants assigned an average of *M* = 3.21 (SD = 2.21) dilemma types per post. First, to determine whether the range of experimenter-supplied dilemma types provided adequate coverage across post content, we sought to ensure that a low percentage of participants were selecting “other” from the list of dilemmas. Indeed, participants chose “other” 0.04% of the time. Second, to determine whether the specific dilemma descriptions were adequately capturing post content, we sought to ensure that participants were providing relatively good “fit” scores to dilemmas. The average fit score across all assigned dilemmas was 3.90 out of 5 (“very well”) (SD = 0.97); 98.3% of posts were assigned at least one dilemma type that fit at least “very well.”

Next, to determine whether the dilemma types were reliably capturing similar post content, we sought to ensure that there was a high degree of *consensus* achieved among raters about which dilemmas applied. For this analysis, we used only posts that had been viewed by three or four raters (*n* = 160) and counted a post as having achieved consensus if it was assigned the same dilemma type by at least two participants. Consensus was achieved on 96.8% of posts. Finally, we sought to ascertain what percentage of the time the person who had written the post (known on Reddit as the “Original Poster” [“OP”]) was the perpetrator (i.e. “person A”) or recipient (“person B”) of the act, or neither. Determining that the OP was mostly the perpetrator rather than the recipient of the act facilitates interpretation of users’ evaluations, since it would indicate that negative evaluations (i.e. “asshole” ratings) typically pertain to the person who committed the act rather than the person who was the recipient of it. The results showed that the OP was person A in 71.5% and person B in 24.5% of the dilemmas assigned (the remaining 4% were indicated as “neither”). This suggests that, as expected, in the majority of cases, the person writing the post was the perpetrator (rather than the recipient) of the act in question. We planned to omit dilemma types identified in <1% of posts, but none fell below this threshold; the least frequently identified dilemma was *secret violation* (3.3% of posts).

### Method 3: Refining the catalog and creating the moral themes

The analysis suggested the initial catalog provided a good fit to the AITA data. On the basis of these results, we made several changes to the labels of the different dilemmas (e.g. we changed “custom violation” to “public transgression”), thereby creating the final version of the catalog (see Table 1). To render this resulting catalog more comprehensible, we assigned the 29 dilemma types to a smaller number of moral themes. Typically, latent variable analysis (e.g. factor analysis or k-means) is used to discover structured or grouping relationships among variables through analysis of cooccurrence or covariance. This approach, however, is poorly suited for the particular task of clustering moral dilemmas, as some dilemmas that bear thematic similarity (e.g. revealing information versus concealing information) may rarely or never cooccur as moral judgments of the same scenario. Thus, while latent variable analysis may yield revealing clusters of correlated dilemmas, we employed a theory-driven approach to create the moral themes. (To view a cluster analysis of the dilemma types, see SOM 3.1 and Fig. S8.)

To identify moral themes in the catalog, we conducted a comprehensive review of existing literature along with a thematic analysis of the dilemma types. First, we assigned dilemma types to categories previously identified in existing taxonomies (15, 20, 31). Where appropriate, we made minor changes to category titles (e.g. we changed “Harm” to “Harm and Offense” to make clear that instances of offense were included in this category; similarly, we changed “Fairness” to “Fairness and Proportionality” to include cases of over- and underreaction). (See SOM 3.1 for associations between our dilemma types and categories from MFT (20).)

Reflecting the growing literature surrounding the role of relationships in shaping moral obligation (15, 17, 26), as well as the high prevalence of such dilemmas in our data, we included a moral theme that involves questions of what people owe to others based on their relationship with them, and includes dilemma types such as *relational omission* (failing to perform a desired behavior) and *relational demand* (wanting someone else to perform a desired behavior) (68). While this theme bears some resemblance to the loyalty foundation in MFT (20), it includes many dilemma types that are better understood as questions of interpersonal obligation than of loyalty per se. For example, the question of whether one is in the wrong for failing to hang up other people's clothes after taking them out of the washing machine (reflected in *relational omission* as shown in Fig. 2) is less a question of loyalty than a question of one's obligations toward the people in one's household. Similarly, a question of whether one is in the wrong for expecting their girlfriend to lint roll their jacket (*relational demand*, Fig. 2) concerns the validity of certain relational expectations rather than questions of allegiance or faithfulness. For this reason, we labeled the theme Relational Obligation. We also included *broken promise* in this category, since making a promise entails creating a contractual relationship between parties (69).

We next sought to identify the most appropriate moral themes for the remaining dilemma types that had not been assigned to one of the three abovementioned themes. One theme we identified that is strangely overlooked in many theories of moral psychology is Honesty, or the set of principles governing questions of truth and falsehood in the sharing of information (70). In this theme, we included seven dilemma types, including *revelation* (telling the hurtful truth), *concealment* (withholding the truth), *misrepresentation* (telling a lie), *secret violation*, and *reporting to authority*. Another theme concerned Social Norms, or the tacit rules that govern behavior in a community or society (71). Past research distinguishes between behaviors that violate rules of morality versus convention (72), yet both play an important role in everyday dilemmas. In this category were included *rudeness*, *public transgressions*, *judgmentalness*, and *privacy violations*. The final moral theme we identified concerned Feelings: a reflection of the fact that, in many posts, emotions emerged not merely as *reactions* to moral events but also as objects of moral evaluation themselves. This includes *private feeling*, as well as *emotional under*- and *overreaction*. The inclusion of Feelings as a moral theme illustrates an important conceptual distinction between our catalog and existing moral taxonomies.

We note that several of the dilemma types could plausibly fit into several moral themes. For example, while we categorized *theft* as a violation of Fairness and Proportionality, it could also be considered an instance of Harm and Offense. Similarly, while we categorized *privacy violation* as an infringement of Social Norms, it could also conceivably be placed in the category of Honesty (since it concerns a potential violation of trust). However, our aim with creating the moral themes was merely to provide an organizational structure that helps group the dilemma types in a readily interpretable way; we concentrate our analyses at the level of dilemma type (as opposed to at the level of moral theme), since such analyses are unaffected by this categorization scheme.

### Method 4: Coding the dataset and creating the training data (“coding study”)

Having created the catalog of everyday dilemmas, we next conducted a large online study in which we recruited human participants to read and code ∼5,000 posts according to the dilemma types identified in the catalog. This study had three purposes. First, it allowed us to verify the validity of the catalog in a larger sample. Second, it allowed us to measure the correlation between negative evaluations produced by the Reddit sample and the study sample, thereby supporting that “asshole” ratings are indeed a generalizable measure of moral evaluation. Finally, it allowed us to create a labeled dataset of many posts assigned to dilemma types, which served as “ground-truth” training data for a language model capable of assigning dilemma types to the remaining 364,071 uncoded posts.

Over 11 rounds of data collection, participants (*n* = 1,227; 476 men, 717 women, and 34 other, *M* age = 33.6, SD = 31.3) were presented with 6–10 dilemmas taken from a randomly selected sample of 5,090 AITA posts. In order to ensure that our distribution of posts adequately reflected those that had been negatively evaluated, as well as those that were controversial, we categorized posts according to whether they were rated NTA, YTA, or controversial and selected equal proportions of posts from among these three categories. Moreover, in order to ensure an even distribution of post engagement, we grouped posts into deciles according to their number of associated comments and selected an equal number of posts from each decile.

After providing informed consent, participants were asked to indicate which dilemma types, if any, each post contained, as well as how well each selected dilemma type described the post, and their final verdict (e.g. NTA or YTA). Our aim was to have each post rated by at least three participants; due to an error in the randomization process, 2% of posts were rated by only two participants; the remainder were rated by three or more participants. Posts rated by two raters were excluded from consensus analysis but included in the training data.

#### Coding results

Participants made a total of 48,171 dilemma type assignments to the 5,090 posts. The average number of dilemmas assigned to each post by each participant was *M* = 2.76 (SD = 2.06); the average number of participants who read each post was *M* = 3.43 (SD = 0.84). As with the pilot study, the results provided by the catalog were comprehensive, accurate, and reliable: participants selected a supplied dilemma type (as opposed to “other”) 99.7% of the time, indicated that at least one dilemma type fit “very well” or “extremely well” for 99% of posts, and reached consensus (i.e. agreed on at least one dilemma type) on 88.2% of posts (see Figs. S1–S3). The average fit assigned across all dilemma types was *M* = 3.92 (SD = 0.96).

To determine the generalizability of the negative evaluations obtained via Reddit users, we also tested the correlation between their evaluations and those given by the online worker sample. The results showed a correlation of *r*(5,088) = 0.59, a large correlation (see Fig. S4). This supports the idea that the evaluations provided by the Reddit sample are generalizable to the broader population.

#### Creating fit scores

To create the training data for each dilemma type, we aggregated participant coded data into a variable known as “fit score.” Fit scores reflect the proportion of “fit points” assigned to each dilemma across each post. Fit points reflect how well each rater thought each assigned dilemma type described each post; dilemma types that were not assigned to a post received fit scores of 0. Fit score allowed us to obtain a “best guess” as to which dilemma types were most prominent in each post relative to all other dilemma types in the catalog. Consider a situation in which a post was assigned the following fit points: rater 1 (privacy violation: 4; secret violation: 5); rater 2 (relational omission: 2); rater 3 (privacy violation: 5). In this case, the total number of fit points is 4 + 5 + 2 + 5 = 16. Privacy violation would receive a fit score of 9/16 = 0.5625; secret violation a fit score of 5/16 = 0.3125; and relational omission a fit score of 2/16 = 0.125. The remaining dilemma types would receive fit scores of 0/16 = 0. This measure allowed us to extract the most likely dilemmas in each post relative to other dilemma types. Because of the relatively small number of selections of “other,” combined with the expected difficulty of the model identifying this multifarious subset of posts in out-of-sample data, we dropped “other” responses from analysis and proceeded only with explicitly labeled dilemmas. In this way, we created a 5,090 × 29 training dataset in which a fit score was assigned to each dilemma type for each post.

### Method 5: Extrapolating from the training data to the remaining dataset

We used techniques from natural language processing and machine learning to extrapolate our fit scores to the full AITA corpus. Overall, our approach was to (i) train several language models on the fit scores in the training data, (ii) determine (via cross-validation) which produced the most accurate and reliable results, and then (iii) apply this trained model to the principal dataset.

We tested and compared four different language models. Our simplest model was a bag-of-words embedding model, which averaged the GloVe word embeddings (73) for each word in a post, to obtain a 300-dimensional post embedding. We then performed a ridge regression on the post embeddings to predict their associated fit scores. The other three models fine-tuned large language models. The goal of fine-tuning is to train the prediction layer appended to the output of the pretrained models. During this process, weights (parameters) of both the pretrained model and the output layer are updated based on the accuracy of the predicted output during batch training with the annotated dataset. The next two models relied on the base BERT (74) and RoBERTa (32) transformer networks. We obtained pretrained versions of these models from the Hugging Face transformers library and fully fine-tuned them on postlevel fit scores. For these models, posts were preprocessed and tokenized using the preexisting model tokenizer in the *tokenizers* library, which splits sentences into a collection of tokens (i.e. words and punctuation) recognizable by the models. Our final model fine-tuned the “Ada” model of GPT-3 (75) obtained from OpenAI accessed via the GPT-3 API, using a batch size of 4 and training the model to 10 epochs, a learning rate of 5e−5 and a weight decay of 0.01.

We evaluated each of these models using 10-fold cross-validation. In this procedure, the dataset is randomly divided into 10 “folds,” or sections. The model is trained on nine of these folds, with one designated as the “hold-out” sample. The trained model then attempts to predict the fit scores for the hold-out sample. This process is repeated ten times with each of the folds as the hold-out sample and the model trained afresh on the remaining nine folds. Across all models, the primary training data consisted of the full post text as input and the fit score [0, 1] as output. Thus, in the test phase, the key performance metric is the correlation coefficient between actual and predicted probability of class membership as a measure of model performance—in other words, the degree to which our trained models’ predictions agreed with the average rating from human raters.

We also compared our model performance to a human benchmark which represents the estimated correlation in fit scores between those reflected in the data and those that would be produced by a single human rater. To calculate this, we selected at random one participant from each post in the coded data and designated this as the “hold-out sample.” We then recalculated the fit scores for each post without this participant and compared them with the ratings in the hold-out sample. The result provides a correlation coefficient for each moral theme reflecting the association between the fit scores provided by the hold-out sample and those reflected in the data.

Model performance metrics for each theme are plotted in Fig. S5. Because our aim in this stage was to compare accuracy across models, it was not necessary to train the model on all 29 dilemma types. Instead, we used fit scores for the six moral themes as training data. (The results are identical if individual dilemma types are used.) As shown in the figure, the human benchmark provided the strongest correlation (average correlation: *r* = 0.58). The most accurate model, RoBERTa, was only slightly less accurate than the human benchmark (average correlation: *r* = 0.51). Moreover, in one moral theme, Relational Obligations, it slightly outperformed the human benchmark (*r* = 0.61 versus *r* = 0.60). Finally, it is noteworthy that the order of accuracy among the six moral themes was roughly the same across all models, including the human benchmark, suggesting that whatever made certain themes harder or easier to identify was true for humans and machines alike. Of course, there will always be some level of disagreement about which moral themes are reflected in a given post; nevertheless, the values obtained in this process suggest that many of the moral themes are identified by the algorithm with an accuracy rivaling that of a human being.

#### Dilemma-type prevalence

Having identified RoBERTa as the highest performing model, we then trained this model on the entire coded dataset of 5,090 posts and used this model to identify the dilemma types in the remaining 364,071 posts. This process was run separately for each of the 29 dilemma types. The result was a 29 × 369,161 matrix in which each cell reflected the predicted fit (“prevalence”) of each dilemma for each post, with theoretical range [0, 1].

#### Dilemma-type presence

In certain cases, it was desirable to identify not just the single most prevalent dilemma type in each post but the nonmutually exclusive list of all prevalent dilemma types in each post, since we postulated that many posts would reflect multiple dilemma types. To do this, it was necessary to convert the predicted fit for each post and each dilemma type into a binary variable reflecting our best guess as to whether the dilemma type was or was not in that post. For this, we first created a “ground-truth” measure based on the coded data using a cascade procedure: first, we assigned to posts all “agreed-upon” dilemma types, where agreed-upon meant having been selected by two or more raters (88% of posts in the sample). If no dilemma types were agreed-upon in a post, then any dilemma types said to fit “extremely well” by a single rater were assigned to the post (8.5% of sample). If no dilemma types were rated as fitting extremely well, then those indicated as fitting “very well” by a single rater were assigned (2.7% of coded sample). Overall, this allowed us to identify the dilemma types present in a total of 4,937 posts (99% of coded sample). We then identified the threshold value for each predicted fit score that maximized the *f*1 value (the harmonic mean between *precision* and *recall*) for each dilemma type.

#### Poster identity

We anticipated that it would be of interest to determine whether the OP was the recipient or the perpetrator of the act described in each post of the principal dataset. Thus, we conducted an additional round of training and extrapolation in which we trained the model on the “identity” scores obtained in the coded sample and then had it predict those same scores in the uncoded data (“predicted identity”).

### Method 6: Identifying relational context (“relationship study”)

We used crowdsourcing and automated text analysis to identify the relationship between the interactants in each post. We recruited a sample of 100 participants on Prolific and, after they provided informed consent, asked them to provide up to 10 words describing specific relationships they had with others (e.g. “mother,” “sister,” “coworker,” etc.). We combined these responses, omitting words that did not indicate a true relationship (e.g. “love bug”) to create a general list of common relationship words. Next, we used text matching to identify which words, if any, appeared in each post, and then combined words that referred to the same relationship (e.g. “mom” = “mother”). Because we were interested in relationships held between the poster and the interactant, we selected only relationship words preceded by the word “my” (e.g. “my neighbor,” “my sister,” etc.). To ensure sufficient size per cell, we selected only those relationship words that appeared in more than 0.1% of posts. A total of 298,632 posts reflecting 41 relationships were identified in this way, which we used for all analysis. The most frequently occurring relationships were “friend” (16% of posts), “mother” (9.5%), and “boyfriend” (9.5%), while the least frequently occurring were “doctor” (1.3%), “buddy” (1.3%), and “therapist” (1.0%; see Fig. S6).

### Method 7: Measuring relational closeness (“closeness study”)

To estimate the average closeness of each relationship, we recruited a separate sample of 100 participants on Prolific and, after they provided informed consent, presented them with the 41 relationships indicated above. We asked them to indicate, “How close is this relationship, on average?” on a sliding scale (0—Not at all close and 100—Extremely close). We calculated the estimated closeness of each relationship by averaging across participant responses (see Fig. S7). We then projected these values onto the full dataset by matching closeness estimates with the relational context detected in each post. We then modeled the association between relational closeness and dilemma prevalence using a hierarchical regression with post and relationship set as random factors to account for nonindependent observations.

### Method 8: Preregistered representative study (“representative study”)

In order to assess the generalizability of our findings, we obtained a sample of everyday dilemmas from a source outside of Reddit and coded it according to the dilemma types identified in the catalog. A sample of 550 participants representative of the US population (simplified US Census), after providing informed consent, was asked to describe in 600 characters or more a personal experience in which they worried they were “in the wrong” (see SOM 2.1–2.2 for materials). Of these, we eliminated 40 due to inadequate response length (<100 characters) or use of ChatGPT to generate a response (generating identical responses about a birthday party), leaving responses from a total of 510 participants (243 men, 258 women, and 9 other, *M* age = 39.4, SD = 12.4). Next, a separate sample of 144 participants (55 men, 60 women, and 29 other/missing, *M* age = 41.2, SD = 12.9) read the descriptions and coded them according to our dilemma type catalog. No participants were excluded from this phase. Note that in our preregistration, we indicated that we would recruit a sample of 250 participants for this coding phase; however, we realized we could improve consistency and save costs by increasing the number of posts participants coded. This led to participants coding more posts per person than we indicated in our preregistration (average of 13.7 posts per participant versus 6–10 as we had originally indicated). As with our previous coding effort, all posts were coded by at least three raters.

In our preregistration (available at https://aspredicted.org/jv8s4.pdf), we predicted that, if the everyday dilemmas collected from the Reddit population were generalizable, and if the catalog was capturing meaningful variations in dilemmas outside of this sample, then three things would be true: first, the average “fit” score of all assigned dilemmas would be >3 out of 5 (“somewhat well”); second, that over 90% of descriptions would be assigned at least one dilemma type fitting at least 4 (“very well”); and third, that the frequency of dilemma types obtained from this sample would be correlated at least *r* = 0.4 with that obtained from the Reddit sample. All three hypotheses were confirmed: the average “fit” of the assigned dilemma types was 3.88 out of 5; the percent of posts assigned at least one dilemma type fitting at least 4 (“very well”) was 98.6%; and the correlation in frequencies between the representative sample and the Reddit sample was *r*(27) = 0.73, *P* < 0.001.

In exploratory analyses, we also tested the degree of consensus obtained by users in the representative sample to further test the validity of the catalog in non-Reddit-specific contexts. The results showed that coders reached excellent levels of consensus in identifying dilemma types, with 90% of descriptions reaching consensus (i.e. having at least one dilemma type agreed upon by at least two coders). This meets or exceeds consensus levels obtained in the AITA data, suggesting that the catalog is capable of reliably coding everyday moral dilemmas outside of AITA.

## Supplementary Material

pgaf119_Supplementary_Data

## Data Availability

All deidentified data are available at the Open Science Repository: https://osf.io/j63dv/? view_only=08fb26a057544a0d9b95c7f61c5d8c95. For additional data and analysis, contact the first author.

## Preprints

This study was posted as a preprint at https://osf.io/preprints/psyarxiv/5pcew.
