# Supplementary material for: A large-scale investigation of everyday moral dilemmas
Source: PNAS Nexus. 2025 May 13;4(5):pgaf119. doi: 10.1093/pnasnexus/pgaf119 (PMC12070388; doi:10.1093/pnasnexus/pgaf119)
Supplement: pgaf119_Supplementary_Data [file pgaf119_supplementary_data.docx]

Supplementary Materials for

**A Large-Scale Investigation of Everyday Moral Dilemmas**

**Authors:** Daniel A. Yudkin, Geoffrey P. Goodwin, Andrew Reece, Kurt Gray, and Sudeep Bhatia

Corresponding author: dyudkin@sas.upenn.edu

**The PDF file includes:**

SOM 1: Figs. S1 to S7

SOM 2: Materials

SOM 3: Supplementary Analyses (and Figure S8)

Tables S1-S2

SOM 4: Survey Questions

[SOM 1: Figures S1-S8 2](#_Toc194607693)

[SOM 2: Materials 9](#_Toc194607694)

[Supplementary Analysis 3.1 11](#_Toc194607695)

[SOM 4.1 Main Survey Questions 18](#_Toc194607696)

# SOM 1: Figures S1-S8


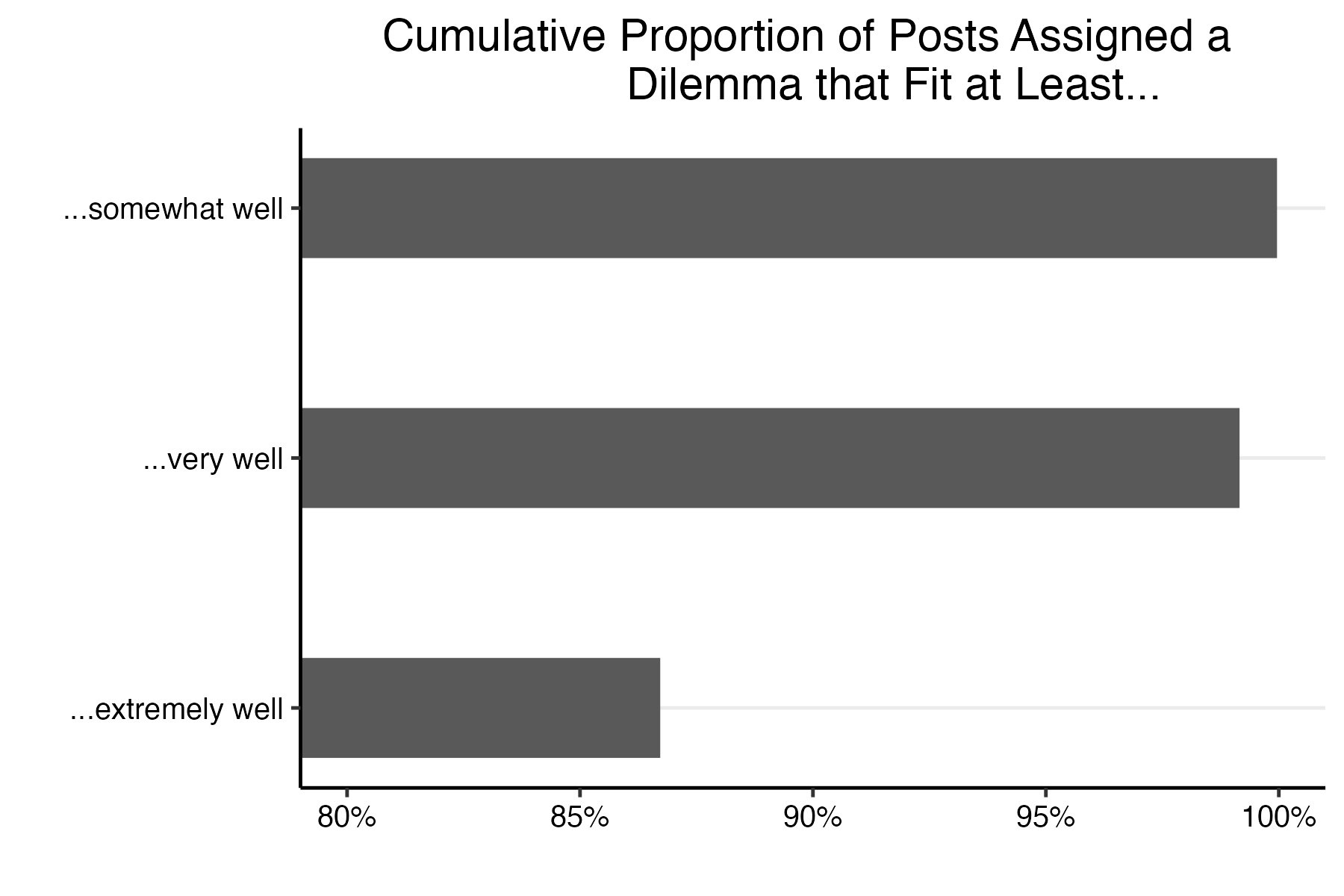


*Figure S1.* Proportion of posts in the coding phase (*n* = 5,090) assigned at least one dilemma type that fit at least somewhat, very, or extremely well. The data show that over 85% of posts were assigned a dilemma type that fit “extremely well” and over 99% were assigned a dilemma type that fit “very well.”


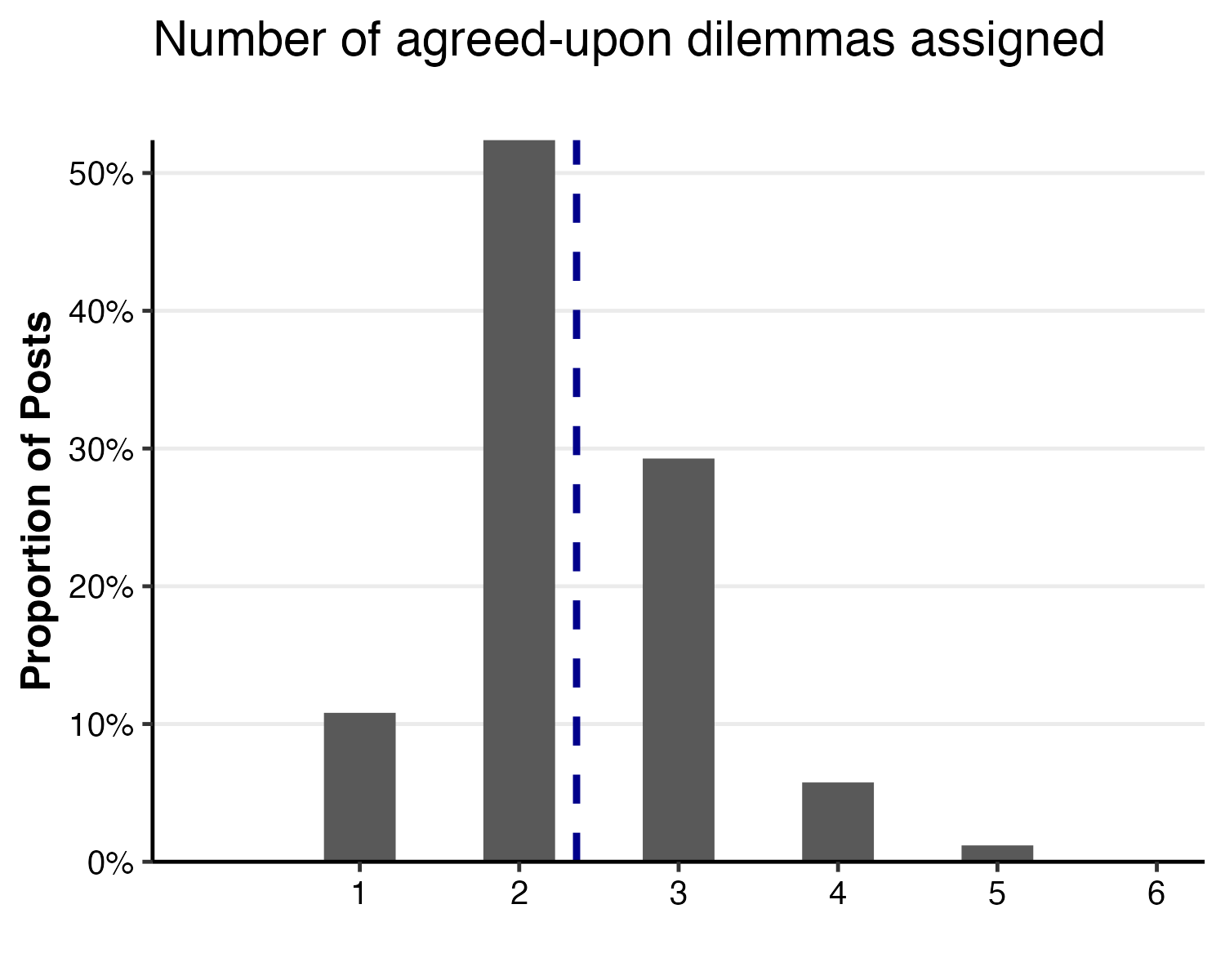


*Figure S2.* Frequency distribution of the proportion of posts (*n* = 5,090) assigned each number of agreed-upon dilemma types by raters (*n* = 1,227), where agreed-upon means that the same dilemma type was selected by two or more participants for a given post. For example, 52.3% of posts were assigned a dilemma type that was agreed upon by two raters. The dotted line represents the mean number of agreed-upon dilemmas assigned per post (*M*  = 2.35).

**
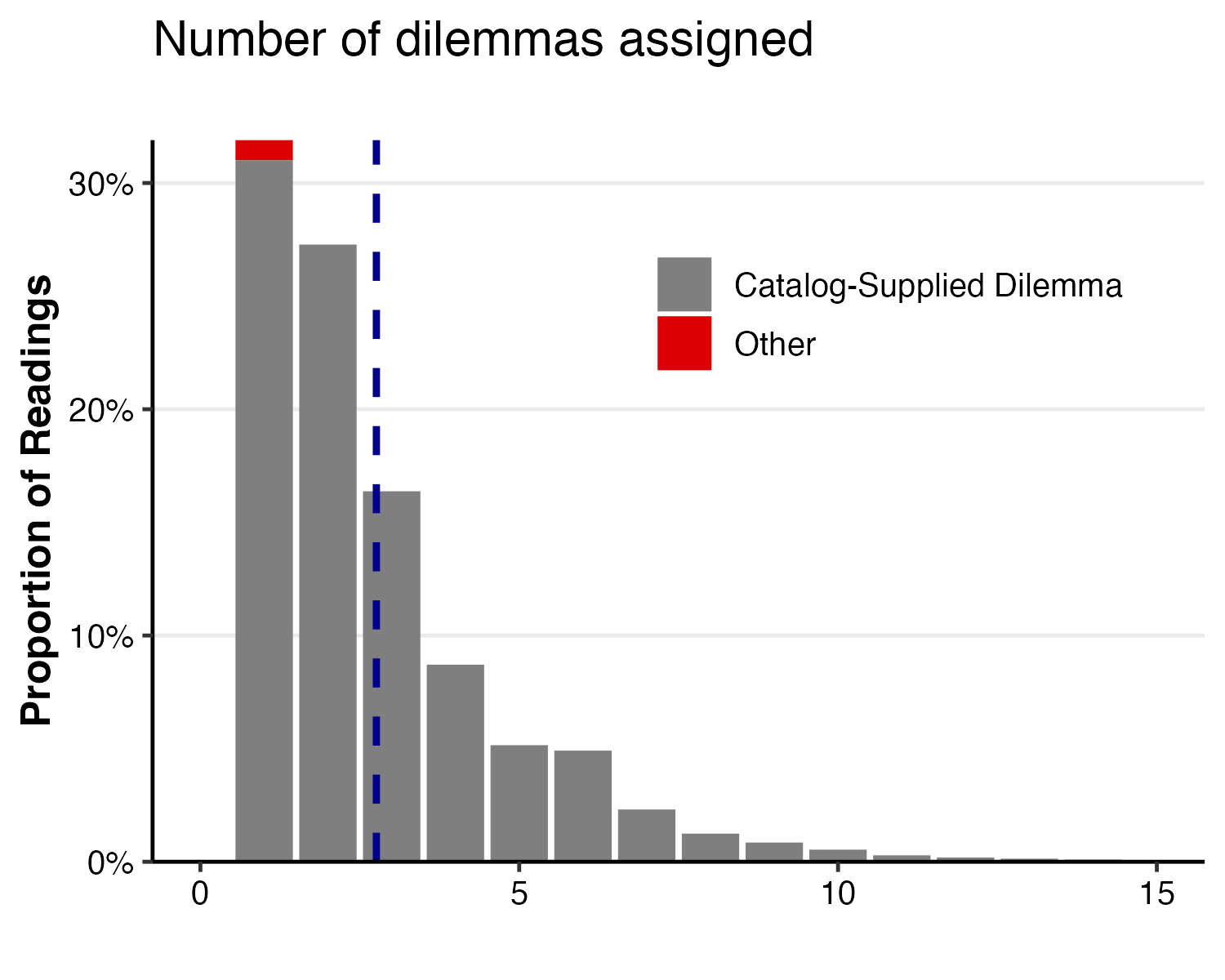
**

*Figure S3.* Frequency distribution of the number of dilemma types assigned per reading of each post by each participant (*n* = 17,469 readings of 5,090 posts). Participants assigned “other” less than 1% of the time. The dotted line represents the mean number of dilemmas assigned per reading (*M*  = 2.76).


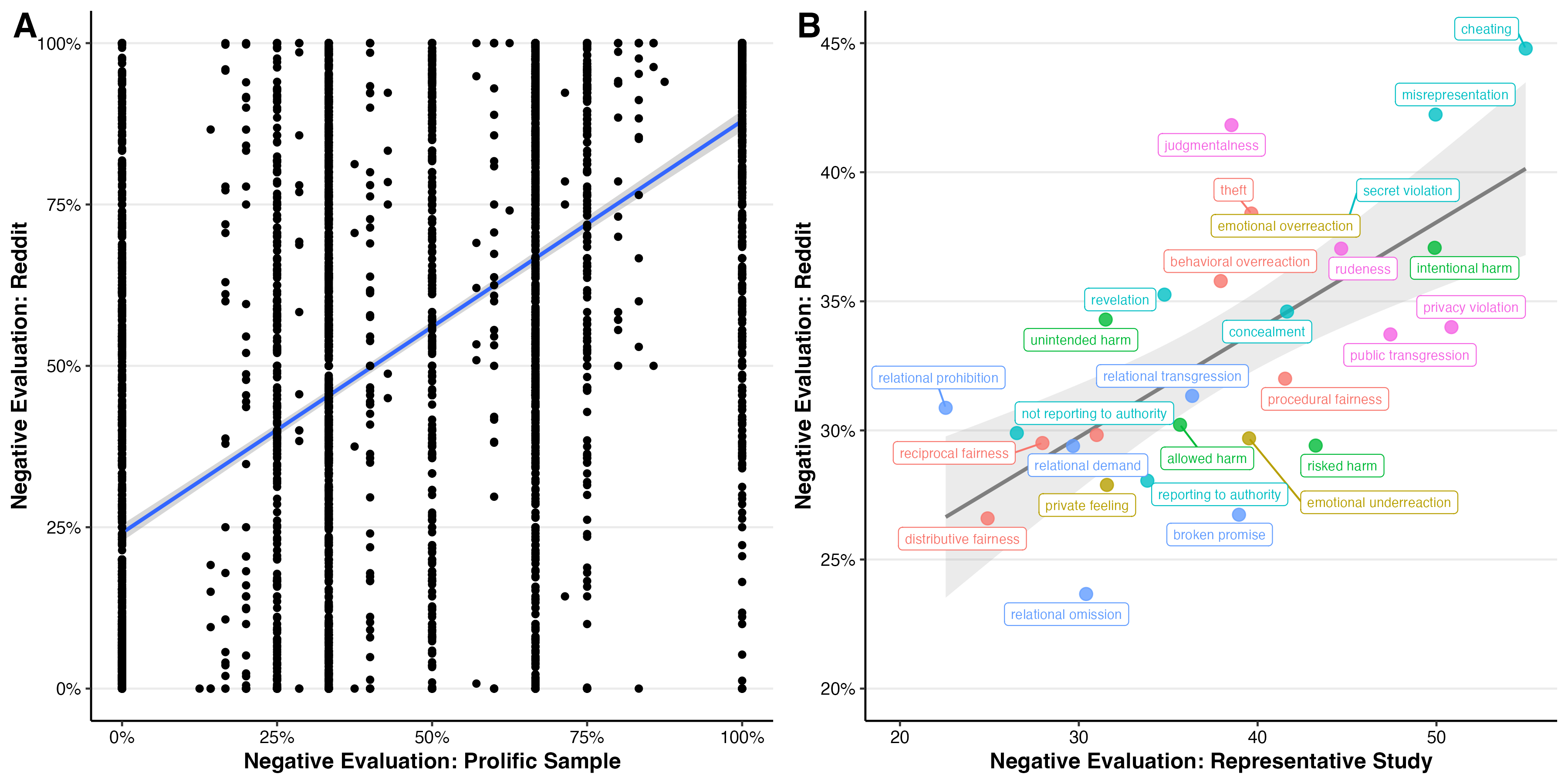


*Figure S4*. Positive associations between negative evaluations (i.e. “asshole” ratings) obtained on Reddit and those obtained from different samples. Panel **A** plots the rates of negative evaluation of each of the 5,090 posts tested in the Coding Study (*n* = 1,227 Prolific participants) against the rates of negative evaluation of those posts obtained on Reddit; *r*(5,088) = .60. Panel **B** plots the average negative evaluation of each dilemma type as measured in the Representative Study (*n* = 110 participants, *n* = 510 dilemmas) against the average negative evaluation of each dilemma type as assessed in the full AITA sample (*n* = 369,161); *r*(27) = .68.


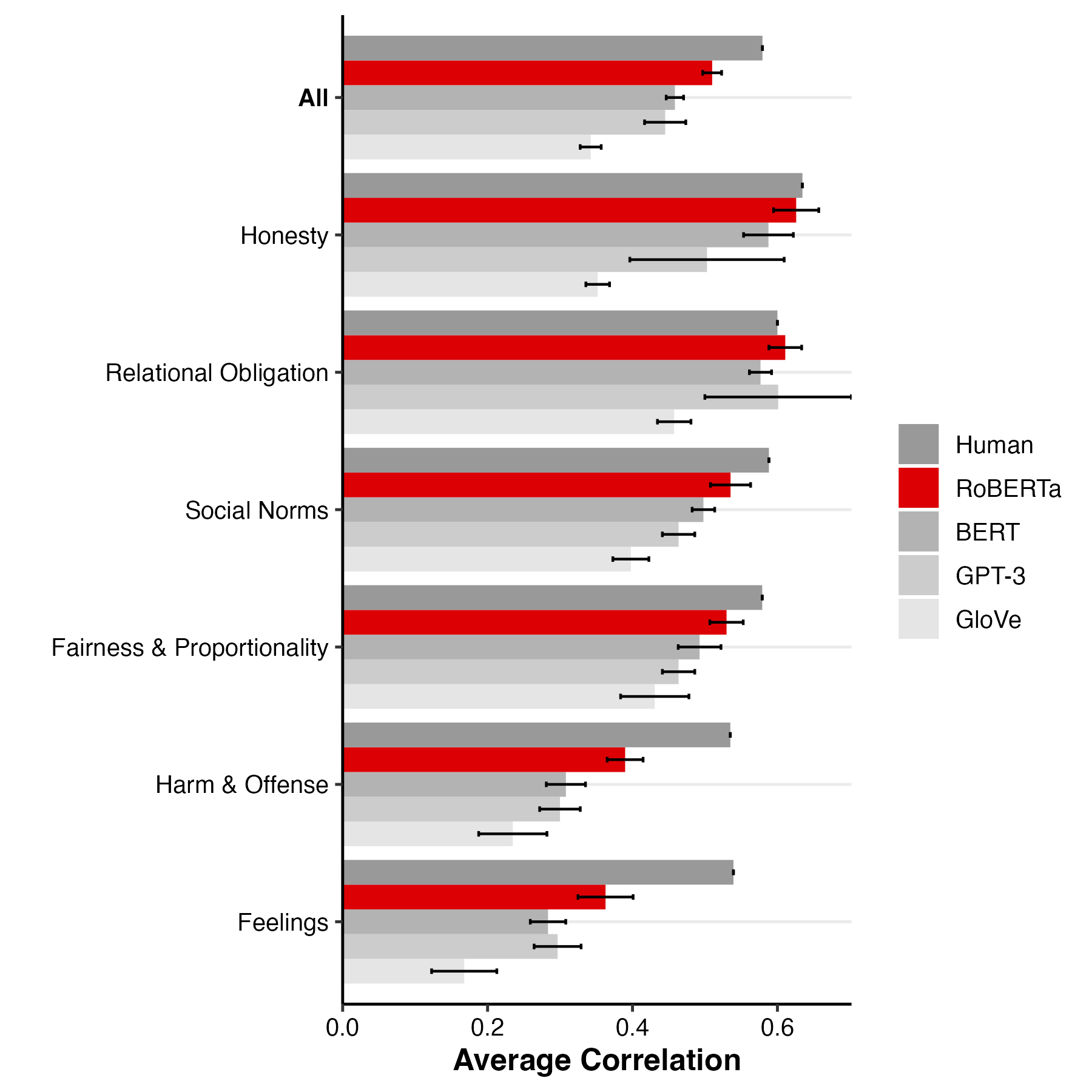


*Figure S5.* Correlations between actual and predicted fit of each moral theme provided by each of the four language models tested, with RoBERTa providing the most accurate predictions after the human benchmark. Error bars = 95% CI.


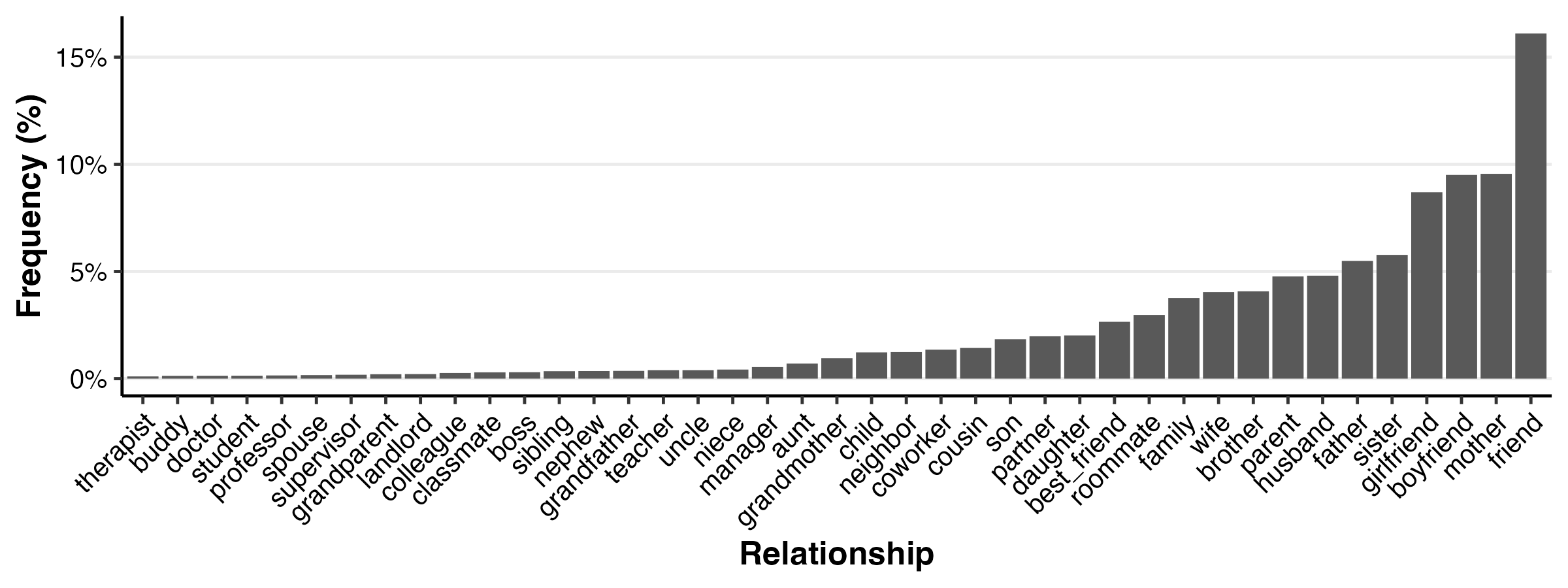


*Figure S6*. Frequencies of relationships extracted from the AITA dataset.


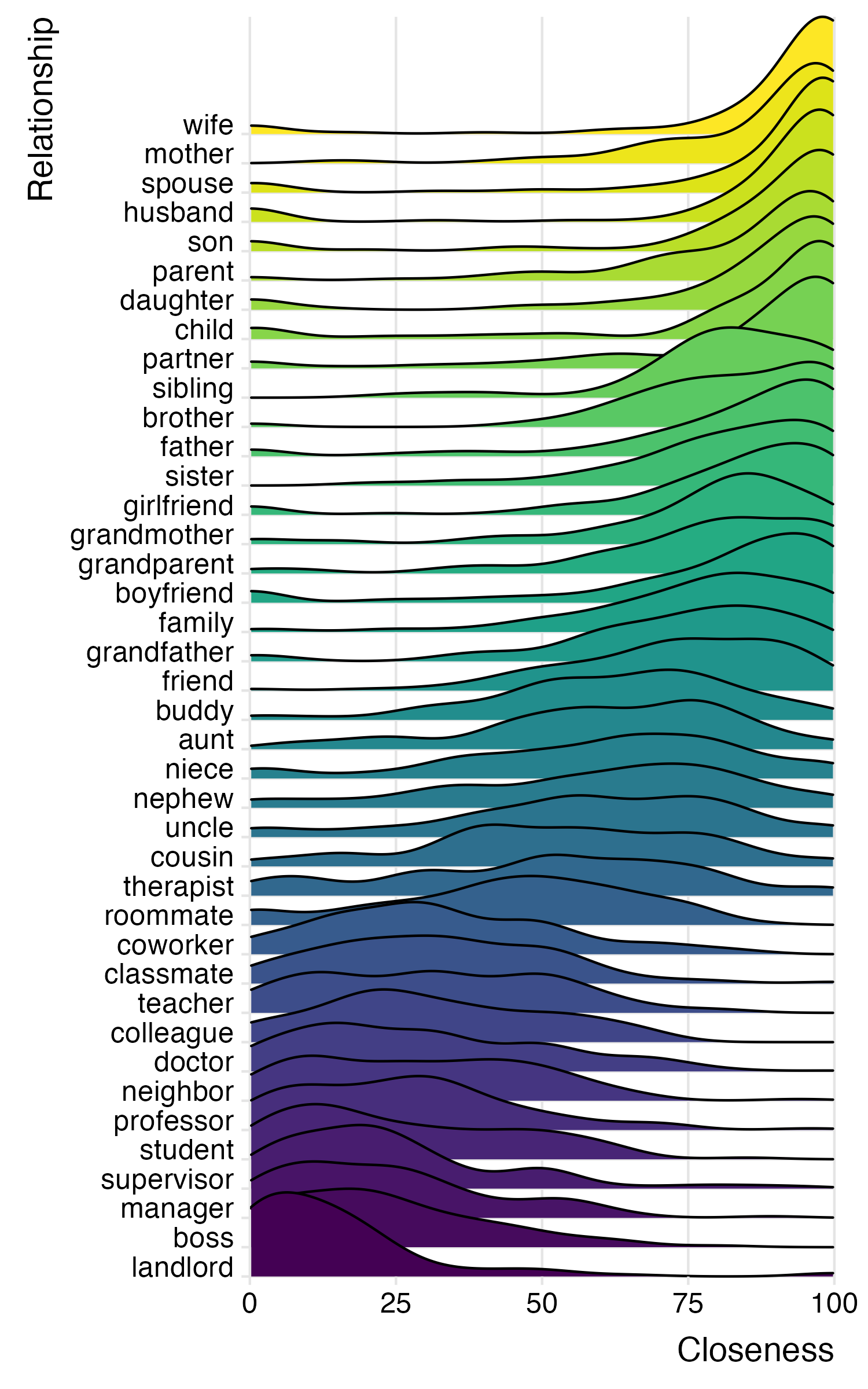


*Figure S7.* Distribution of closeness rates of each relationship obtained from separate online sample (*N* = 100).

# SOM 2: Materials

**SOM 2.1: Coding Study**

**[Dilemma assignment]**

*Please select the category or categories that best fit the actions being considered or described.*

*Select as many as you think apply.*

[Participants are presented names and descriptions of 29 dilemma types, along with “Other (please describe)”]

**[Fit]**

*How well do the category or categories fit?*

*Please indicate how well the categori(es) you selected describe the actions considered or depicted in the post.*

*1 – Not at all well*

*2 – A little well*

*3 – Somewhat well*

*4 – Very well*

*5 – Extremely well*

**[Identity]**

*Who's who?*

*For this question, we need your help identifying who in the post the descriptions refer to. 
For each category you selected, please indicate whether "Person A," "Person B," or "Someone else" refers to the person who wrote the post.*

*"The person who wrote the post is...."*

- *Person A*
- *Person B*
- *Someone else*

**[Evaluation]**

*Your turn. Now we would like you to give your verdict. On the "Am I the Asshole" subreddit, users indicate one of four evaluations of the person who wrote the post (see below).

What do****you****think about the person who wrote this post?*

- *“Not the asshole” (NTA)*
- *“You’re the asshole” (YTA)*
- *“No assholes here” (NAH)*
- *“Everyone sucks here” (ESH)*

**SOM 2.2: Representative Study**

**[Instructions]**

*We would like you to tell us about a personal experience you've had that caused you to worry about whether you were****in the wrong****.

For instance, you might have done (or not done) something that caused one or more people to react with surprise, hurt, annoyance, disappointment, unfriendliness, anger, or some other negative response. This, in turn, caused you to worry or feel uncertain about whether you were in the wrong.

In at least 600 characters (100-150 words), please describe a time when something like this happened.*

**[Evaluation]**

*How wrong do you think the writer's actions were in this situation?*

*[Sliding scale: 0 – “Not at all wrong”; 100 – “Very wrong”]*

Supplementary Analysis 3.1**: K-means analysis**

In order to identify groups of co-occurring dilemmas, we performed a k-means cluster analysis on the prevalence values of each dilemma type across all posts. Prior to performing the analysis the optimal number of clusters *k* must be identified. An analysis of within-cluster sum of squares for values of *k* ranging from 2 to 10 yielded no obvious cut point. Given recent advances in moral psychology identifying 6 clusters of moral concern (Atari et al., 2023), we specified *k* = 6 clusters. (Notably, a principal components analysis also found 6 principal dimensions of the data.) We then plotted the clusters on a projection of the first two principal components of the data (see Figure S9 below).

The results highlighted some expected and unexpected associations. Not surprisingly, *relational omission* and *relational demand* occurred in the same cluster, as did all three forms of fairness: *reciprocal*, *distributive*, and *procedural*. Many forms of dishonesty, including defection, misrepresentation, and *secret violation* occurred in one cluster, which also contained instances of *reporting to authority* and *privacy violation*. Finally, *public transgressions* occurred in a cluster with *politeness* and *unintended harm*. Unexpectedly, a large cluster of data emerged containing all the Feelings-related dilemmas (e.g., *private feeling* and *emotional overreaction*), as well as *intentional harm*, *relational transgression*, and *relational prohibition*. Moreover, the dilemma of *concealment* appeared in its own cluster. Overall, this approach yielded clusters that confirms many commonsense expectations about which dilemma types would co-occur but also yields some unexpected findings that highlight fruitful avenues for further investigation.

*Figure S8*. Visualization of a k-means clustering algorithm of dilemma prevalence, with each dilemma plotted over the first two principle components of the data (N = 369,161).

**Supplementary Analysis 3.2: Moral Foundations Theory and Everyday Dilemmas.** Previous research has relied on moral foundations theory to identify moral themes in natural language(Hoover et al., 2020; Hopp et al., 2020) . MFT identifies five overarching moral concerns: care/harm, fairness/cheating, loyalty/betrayal, authority/subversion, and purity/degradation. Thus, it was of interest to determine how moral themes identified in the AITA dilemma catalog matched or deviated from those identified in MFT. To do this, we first extracted the relevant moral dimensions from each post in the principal dataset using the Extended Moral Foundations Dictionary (eMFD)(Hopp et al., 2020). We then tested for correlations between the prevalence in each post of each dilemma type in our catalog and the prevalence of each moral foundation.

Our hypotheses were as follows. First, we expected that clearly overlapping themes in both taxonomies would exhibit the strongest correlations. For instance, we expected the different types of fairness (e.g., *distributive*, *procedural*, and *reciprocal*) as well as *cheating* to correlate robustly with the fairness/cheating foundation in the eMFD. We expected the different forms of harm (e.g., *intentional*, *unintended*) to correlate with the care/harm foundation. We expected dilemma types relating to *reporting* and *not reporting to authority* to correlate with the authority/subversion foundation. And we expected dilemma types relating to relational obligations (e.g., *relational demand* and *relational omission transgression*) to be correlated with loyalty. Finding these correlations would support both theories, suggesting that both approaches are identifying expected patterns in the data. By contrast, we were agnostic about how less clearly aligned dilemma types would correlate with the different foundations. For example, we did not have strong *a priori* predictions about how the various honesty-related dilemmas (e.g., *misrepresentation*, *concealment*) would be associated with Moral Foundations; likewise with such dilemmas as *judgmentalness* and *impoliteness*. Finally, we did not have strong predictions about how the sanctity/purity foundation would correlate with the different dilemmas.

***Results***. A full breakdown of the associations is displayed in Figure S4. Because of the large sample size, even very small correlations (e.g., *r* = .01) are statistically significant. To assess which foundation is most strongly correlated with each dilemma type, we highlight the foundation with the largest positive correlation for each dilemma type. The results largely conform to predictions but deviate in some ways. For example, as expected, for all forms of fairness, (*distributive*, *procedural*, and *reciprocal*), as well as *cheating*, the most strongly associated foundation is fairness/cheating (*r*  = .29, *r*  = .20, *r*  = .27, and *r*  = .12, respectively). Similarly, for dilemmas involved *risked harm* and *allowed harm*, the most strongly associated foundation is care/harm (*r* = .14 and *r* = .17). And *reporting to authority* is most strongly associated with authority/subversion (*r* = .11). These patterns support the idea that the categories identified in each model are associated with one another in expected ways. On the other hand, certain unexpected associations also emerged, which provide additional insight as to the moral makeup of certain everyday dilemmas. For example, the dilemmas most strongly associated with sanctity/degradation include *judgmentalness* (*r*  = .16), *relational prohibition* (*r*  = .16), and *relational transgression* (*r* = .18). We speculate that this reflects the fact that these dilemmas all reflect a form of disrespect. Moreover, *unintended harm* did not positively correlate with any of the moral foundations. Furthermore, there are areas where the relationship is less clear. For example, dilemmas concerning *rudeness* do not positively correlate with any of the moral foundations; similarly, *privacy violations* do not show any strong relationships with moral foundations extracted via the dictionary method. This reveals areas of convergence and divergence between these methods, thereby highlighting the complementary strengths of these different approaches in mapping the moral terrain.


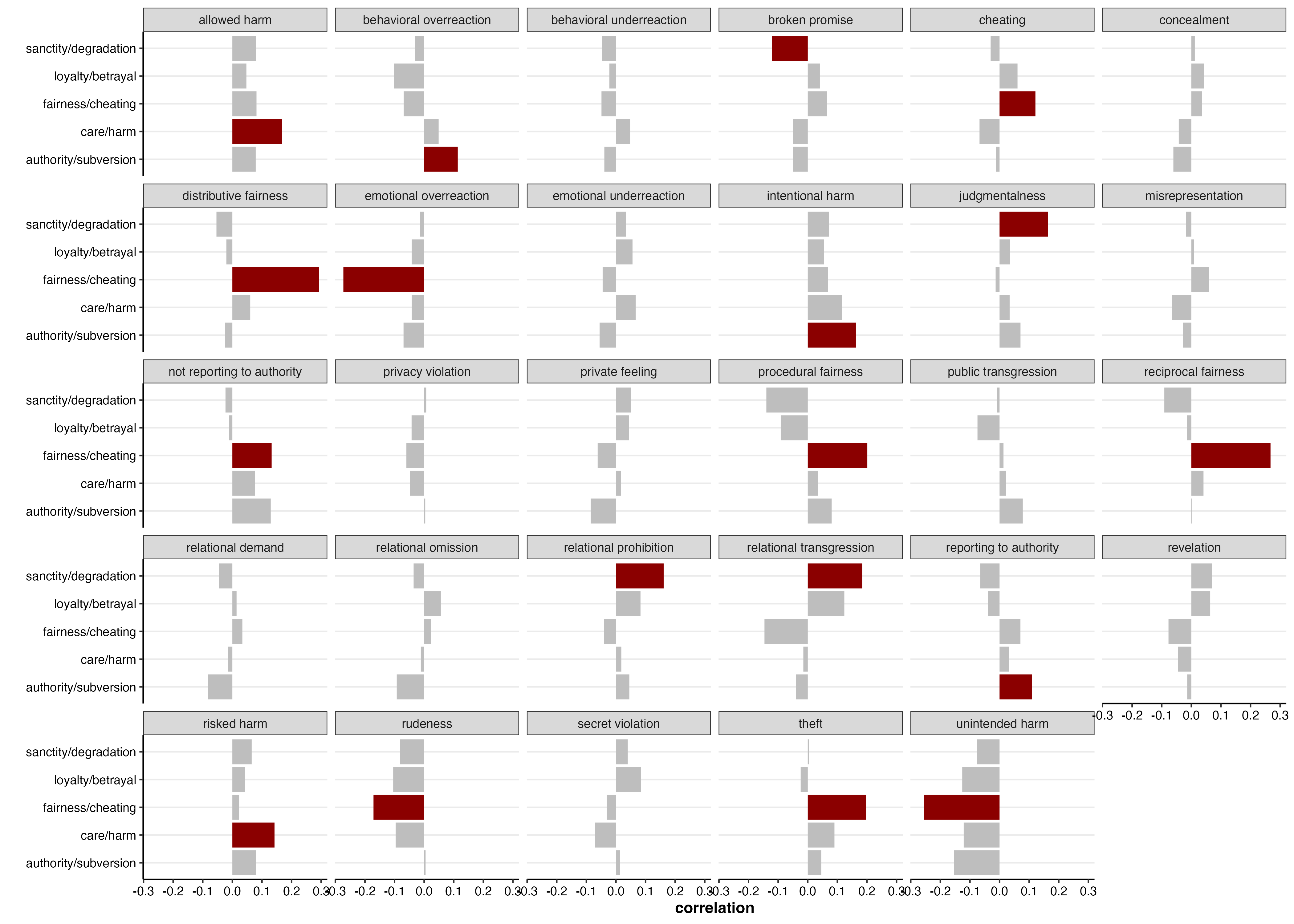


*Figure S9.* Correlations between categories in the MFD and the AITA dilemma catalog. In this graph, red bars correspond to the highest magnitude correlation within each dilemma type. Only correlations where *r* > .1 are highlighted.

**Supplementary Analysis 3.3: Everyday Dilemmas and Relational Models Theory (RMT).** In the main paper, we examined how the prevalence of different dilemma types varies according to different relationships and interpersonal closeness. Yet closeness is not the only dimension on which relationships may vary. Past research suggests that social interactions may be divided into four different models: Communal Sharing, where group members pool resources and support each other without tracking individual contributions; Authority Ranking, where individuals rights and responsibilities to one another are organized by a rank ordering of their power, status, or role; Equality Matching, where individuals aim for fair exchanges and contributions; and Market Pricing, where interactions are driven by transactions, costs, and benefits (Fiske, 1992). More recently, researchers have examined how these different relational contexts may impact the prevalence of different moral foundations (Simpson et al., 2016). They have found that people’s evaluation of different moral infractions differs according to relational context. For example, violations of respect/authority are rated most negatively in the context of Authority Ranking relationships (i.e., a student-professor relationship), and violations of loyalty are considered most wrong in the context of Communal Sharing relationships (i.e., between siblings).

Here we expand on this research to explore how different relational models are associated with the prevalence of different everyday dilemmas. We identified words describing each of the different relational models from past research, then compared the prevalence of each dilemma in posts containing those relational models.

**Table S1**. *Words used to identify each of the relational models*

| **Relational Model** | **Example Relationship** | **Matched words** |
| --- | --- | --- |
| Communal Sharing | Sibling | “brother,” “sister,” “sibling” |
| Authority Ranking | Student-professor | “student,” “teacher,” “professor,” “prof” |
| Equality Matching | Housemates | “roommate,” “housemate” |
| Market Pricing | Customer-salesperson | “customer,” “salesman,” “client,” “saleswoman,” “salesperson” |

We expected to corroborate many of the patterns identified in past research. For example, given that previous research found that the loyalty/betrayal foundation is most prevalent in the Communal Sharing context, we expected that Relational Obligations would similarly be most prevalent in Communal Sharing relationships. Similarly, since the fairness/cheating foundation is most prevalent in Market Pricing relationships, we expected that dilemma types related to fairness would be most prevalent in these relationships.

We also expected certain patterns not yet observed in existing research. For example, because Market Pricing relationships are regulated by social norms regarding appropriate conduct in transactions, we expected *politeness* dilemma types to be most prevalent in these relationships. In addition, we expected *reporting to authority* to be most prevalent in Authority Ranking relationships. Finally, because secrets are typically kept and shared between close others, we expected *secret violations* to be most prevalent in Communal Sharing relationships.

***Results*.** We report the prevalence of each dilemma type within each relational model in Table S2. As seen in the table, the results corroborate many of our predictions. For example, as predicted, we find that several forms of fairness (*procedural* and *reciprocal*) are most prevalent in Market Pricing relationships. And many forms of relational obligation (e.g., *omission, transgression*, and *prohibition*) are all most prevalent in Communal Sharing relationships, as is *secret violation*. Moreover, as expected, dilemma types involving impoliteness were most prevalent in Market Pricing relationships, supporting the idea that norms around appropriate decorum are prevalent in such settings.

On the other hand, several of our predictions were not borne out. For example, *reporting to authority* was most prevalent in Market Pricing relationships, rather than Authority Ranking relationships. One possible reason for this is that moral dilemmas at the workplace (that is, those that may elicit the Market Pricing designation) also tend to include mentions of authority figures (e.g., manager, boss), thereby creating an association between settings where market forces are involved and those entailing a hierarchical relationship. In addition, *distributive fairness* was most prevalent in Equality Matching relationships rather than Market Pricing relationships. We speculate that this is because distributive fairness pertains to allocating resources between equals, as is the case in Equality Matching. Finally, *relational demand* was most prevalent in Equality Matching relationships as opposed to Communal Sharing relationships. One possible reason for this is that dilemmas that occur in relationships governed by equal partnerships (e.g., roommate relationships) may be more likely to entail interpersonal requests or demands.

In addition, there were several findings that were not hypothesized but that nevertheless help shed light on the nature of the everyday dilemmas and the relational contexts in which they occur. For example, all emotion-related dilemmas are most prevalent in Communal Sharing relationships, suggesting that the appropriateness of different emotional responses might be most in question in these sorts of interactions. Interestingly, the relational context in which many of the Honesty-related dilemmas were most prevalent was Authority Ranking. It is possible this finding is due to the fact that certain types of honesty are most relevant in hierarchical relationships; on the other hand, this finding may be the result of the fact that Authority Ranking was operationalized in the “professor-student” relationship, which may be more likely to pertain to honesty for reasons that have nothing to do with authority (e.g., questions around honesty and integrity with, say, written work and exams). Finally, we see that both *theft* and *privacy* *violation* are both most prevalent in Equality Matching relationships, highlighting the fact that dilemmas concerning personal property and privacy are most likely to emerge in roommate relationships (the relationship in which Equality Matching was instantiated). Overall, these results present a useful additional perspective from which to understand how moral dilemmas emerge in different social contexts.

**Table S2**. Mean prevalence of each dilemma type in each Relational Model. The Relational Model wherein each dilemma type is most prevalent is indicated in bold, standard deviation in parentheses.

|  | **Relational Model** | | | |
| --- | --- | --- | --- | --- |
| **Dilemma Type** | **Authority Ranking** | **Communal Sharing** | **Equality Matching** | **Market Pricing** |
| **Fairness** |  |  |  |  |
| Behavioral overreaction | 0.0545 (0.023) | 0.0543 (0.024) | 0.0589 (0.023) | **0.0679 (0.029)** |
| Behavioral underreaction | 0.0413 (0.018) | **0.0459 (0.02)** | 0.0423 (0.019) | 0.0401 (0.017) |
| Distributive fairness | 0.0584 (0.058) | 0.0494 (0.048) | **0.0789 (0.078)** | 0.044 (0.034) |
| Procedural fairness | 0.0612 (0.039) | 0.0431 (0.02) | 0.0578 (0.03) | **0.0816 (0.056)** |
| Reciprocal fairness | 0.0379 (0.031) | 0.0347 (0.026) | 0.0397 (0.031) | **0.043 (0.036)** |
| theft | 0.0224 (0.03) | 0.0213 (0.032) | **0.0302 (0.04)** | 0.0268 (0.032) |
| **Emotions** |  |  |  |  |
| Emotional overreaction | 0.0586 (0.023) | **0.0641 (0.025)** | 0.0571 (0.022) | 0.0549 (0.022) |
| Emotional underreaction | 0.0428 (0.02) | **0.0486 (0.024)** | 0.0412 (0.02) | 0.0403 (0.019) |
| Private feeling | 0.0557 (0.034) | **0.0578 (0.037)** | 0.0523 (0.033) | 0.0482 (0.029) |
| **Harm** |  |  |  |  |
| Allowed harm | 0.0324 (0.017) | 0.0378 (0.018) | 0.0367 (0.018) | 0.0376 (0.019) |
| Intentional harm | 0.0635 (0.039) | 0.0628 (0.039) | 0.0592 (0.033) | **0.0648 (0.037)** |
| Risked harm | 0.062 (0.029) | 0.0601 (0.028) | **0.0633 (0.034)** | 0.0582 (0.028) |
| Unintended harm | 0.0925 (0.042) | 0.0904 (0.039) | 0.0878 (0.036) | **0.0975 (0.045)** |
| **Honesty** |  |  |  |  |
| Concealment | **0.0499 (0.05)** | 0.0458 (0.051) | 0.0413 (0.044) | 0.0465 (0.042) |
| Cheating | **0.0275 (0.019)** | 0.0216 (0.015) | 0.0251 (0.016) | 0.0233 (0.017) |
| Misrepresentation | 0.0366 (0.032) | 0.0317 (0.027) | 0.0287 (0.025) | **0.0388 (0.031)** |
| Revelation | **0.0603 (0.037)** | 0.0593 (0.034) | 0.0506 (0.03) | 0.0554 (0.032) |
| Not reporting to authority | 0.0277 (0.018) | 0.0209 (0.014) | 0.018 (0.013) | **0.0345 (0.02)** |
| Reporting to authority | 0.044 (0.047) | 0.0302 (0.027) | 0.0355 (0.034) | **0.0488 (0.051)** |
| Secret violation | 0.0194 (0.02) | **0.0199 (0.022)** | 0.0169 (0.018) | 0.0186 (0.018) |
| **Relational Obligation** |  |  |  |  |
| Broken promise | 0.0505 (0.047) | 0.0413 (0.036) | **0.0641 (0.053)** | 0.0367 (0.038) |
| Relational demand | 0.0718 (0.052) | 0.0832 (0.044) | **0.0834 (0.046)** | 0.0523 (0.039) |
| Relational omission | 0.0781 (0.06) | **0.0977 (0.065)** | 0.0787 (0.053) | 0.0543 (0.043) |
| Relational prohibition | 0.0364 (0.032) | **0.0495 (0.037)** | 0.0455 (0.041) | 0.027 (0.027) |
| Relational transgression | 0.0669 (0.03) | **0.0928 (0.031)** | 0.0799 (0.028) | 0.0535 (0.025) |
| **Social Norms** |  |  |  |  |
| judgment | **0.0521 (0.032)** | 0.0516 (0.029) | 0.0449 (0.027) | 0.0513 (0.03) |
| politeness | 0.0734 (0.049) | 0.0719 (0.042) | 0.0682 (0.038) | **0.0913 (0.062)** |
| Privacy violation | 0.0429 (0.036) | 0.0438 (0.038) | **0.0542 (0.04)** | 0.0442 (0.037) |
| Public transgression | 0.07 (0.049) | 0.0509 (0.033) | 0.0497 (0.027) | **0.0908 (0.055)** |

**References**

Atari, M., Haidt, J., Graham, J., Koleva, S., Stevens, S. T., & Dehghani, M. (2023). Morality beyond the WEIRD: How the nomological network of morality varies across cultures. *Journal of Personality and Social Psychology*.

Fiske, A. P. (1992). The four elementary forms of sociality: Framework for a unified theory of social relations. *Psychological Review*, *99*(4), 689.

Hoover, J., Portillo-Wightman, G., Yeh, L., Havaldar, S., Davani, A. M., Lin, Y., Kennedy, B., Atari, M., Kamel, Z., Mendlen, M., Moreno, G., Park, C., Chang, T. E., Chin, J., Leong, C., Leung, J. Y., Mirinjian, A., & Dehghani, M. (2020). Moral Foundations Twitter Corpus: A Collection of 35k Tweets Annotated for Moral Sentiment. *Social Psychological and Personality Science*. https://doi.org/10.1177/1948550619876629

Hopp, F. R., Fisher, J. T., Cornell, D., Huskey, R., & Weber, R. (2020). The extended Moral Foundations Dictionary (eMFD): Development and applications of a crowd-sourced approach to extracting moral intuitions from text. *Behavior Research Methods*, 1–23. https://doi.org/10.3758/s13428-020-01433-0

Simpson, A., Laham, S. M., & Fiske, A. P. (2016). Wrongness in different relationships: Relational context effects on moral judgment. *Journal of Social Psychology*, *156*(6), 594–609. https://doi.org/10.1080/00224545.2016.1140118

# SOM 4.1 Main Survey Questions

[Following instructions, training, and 2 practice rounds]

On the next page, you will begin evaluating "Am I in the Wrong?" stodries. You will evaluate 10 posts in all.

For each story, you will be asked:

- ☐ Which category or categories best describe the story?
- ☐ How well does each category fit?
- ☐ Who is the story is about?
- ☐ Do you believe the writer was in wrong?

Remember:

- ☐ You may select as many categories as you wish
- ☐ However, if one seems best, please select only one
- ☐ If no category fits, please select 'other' at the bottom of the category list and provide your own explanation

End of Block: read_begin

What is happening in this post?

Please select the category or categories that best fit the actions being considered or described.

Don't rush! Please take your time and make sure your response is accurate.

Select as many as you think apply (but one is fine!).

▢ Relational Omission – Person A may not be performing a behavior that Person B wants or expects them to do in the context of a relationship (1)

▢ Relational Transgression – Person A may be performing or seeking to perform a behavior that Person B disapproves of in the context of a relationship (2)

▢ Relational Demand – Person A may be expecting Person B to perform a given behavior, or feeling upset that behavior was not performed in the context of a relationship (3)

▢ Relational Prohibition – Person A may be attempting to prevent Person B from performing a behavior in the context of a relationship (4)

▢ Broken Promise – Person A may be going back on a commitment (5)

▢ Private Feeling – Person A may be experiencing an inappropriate or excessive emotion or desire that is unknown to others (6)

▢ Emotional Underreaction – Person A may be feeling or displaying too little of a desirable emotion (7)

▢ Emotional Overreaction – Person A may be displaying too much of an undesirable emotion (8)

▢ Procedural Fairness – Person A may not be adhering correctly to a principle or procedure (9)

▢ Distributive Fairness – Person A may not be making an appropriate allocation of resources, or be contributing their fair share (10)

▢ Reciprocal Fairness – Person A may be failing to sufficiently compensate Person B for an initial act (11)

▢ Behavioral Underreaction – Person A may be underreacting in response to Person B's behavior or predicament (12)

▢ Behavioral Overreaction – Person A may be overreacting in response to Person B's behavior or predicament (13)

▢ Theft/Property – Person A may be taking something that doesn't belong to them (14)

▢ Concealment – Person A may be concealing information (15)

▢ Revelation – Person A may be causing harm, offense, or other negative emotions by telling the truth (16)

▢ Misrepresentation – Person A may be being deliberately dishonest (17)

▢ Reporting to Authority – Person A may be reporting Person B to authority (18)

▢ Not Reporting to Authority – Person A may be declining to report Person B to authority (19)

▢ Secret Violation – Person A may be divulging Person B's secret (20)

▢ Defection – Person A may be cheating in a cooperative or trusting situation (21)

▢ Public Transgression – Person A may be publicly violating a social norm or convention (22)

▢ Privacy Violation – Person A may be violating Person B's privacy (23)

▢ Politeness – Person A may be being rude to Person B (24)

▢ Judgment – Person A may be passing judgement on Person B (25)

▢ Unintended – Person A may be accidentally harming, offending, or otherwise negatively impacting Person B (26)

▢ Intentional – Person A may be purposely harming, offending, or otherwise negatively impacting Person B (27)

▢ Allowed – Person A may be failing to prevent harm, offense, or other negative consequences from befalling Person B (28)

▢ Risked – Person A may be risking causing harm, offense, or other negative consequences on Person B (29)

▢ Other (please describe): _______________________

## How well do the category or categories fit?

Please indicate how well the categori(es) you selected describe the actions considered or depicted in the post.

1 - Not at all well, 2 - A little well, 3 - Somewhat well, 4 - Very well, 5 - Extremely well

## Who's who?

For this question, we need your help identifying who in the post the descriptions refer to.

For each category you selected, please indicate whether "Person A," "Person B," or "Someone else" refers to the person who wrote the post.

"The person who wrote the post is...."

Options: Person A, Person B, Someone else

## How wrong do you think the writer's actions were in this situation?

Scale: 0 (Not at all wrong) to 100 (Very wrong)

If you'd like to explain your answer, you may do so in the space below.

________________________________________________________________

________________________________________________________________

________________________________________________________________

## Demographic Questions

What is your age?

________________________________________________________________

What is your gender?

- Man/Male
- Woman/Female
- Other/fluid

What is your race/ethnicity?

- White
- Black or African American
- American Indian or Alaska Native
- Asian
- Native Hawaiian or Pacific Islander
- Hispanic/Latino
- Other

What is your current mood?

- Very positive
- Somewhat positive
- Neutral
- Somewhat negative
- Very negative

What is your political orientation?

- Very liberal
- Somewhat liberal
- Moderate
- Somewhat conservative
- Very conservative
